# Genetic silencing of olivocerebellar synapses causes dystonia-like behaviour in mice

**DOI:** 10.1038/ncomms14912

**Published:** 2017-04-04

**Authors:** Joshua J. White, Roy V. Sillitoe

**Affiliations:** 1 Department of Pathology and Immunology, Baylor College of Medicine, Houston, Texas 77030, USA; 2 Department of Neuroscience, Baylor College of Medicine, Houston, Texas 77030, USA; 3 Jan and Dan Duncan Neurological Research Institute of Texas Children's Hospital, 1250 Moursund Street, Suite 1325, Houston, Texas 77030, USA; 4 Program in Developmental Biology, Baylor College of Medicine, Houston, Texas 77030, USA

## Abstract

Theories of cerebellar function place the inferior olive to cerebellum connection at the centre of motor behaviour. One possible implication of this is that disruption of olivocerebellar signalling could play a major role in initiating motor disease. To test this, we devised a mouse genetics approach to silence glutamatergic signalling only at olivocerebellar synapses. The resulting mice had a severe neurological condition that mimicked the early-onset twisting, stiff limbs and tremor that is observed in dystonia, a debilitating movement disease. By blocking olivocerebellar excitatory neurotransmission, we eliminated Purkinje cell complex spikes and induced aberrant cerebellar nuclear activity. Pharmacologically inhibiting the erratic output of the cerebellar nuclei in the mutant mice improved movement. Furthermore, deep brain stimulation directed to the interposed cerebellar nuclei reduced dystonia-like postures in these mice. Collectively, our data uncover a neural mechanism by which olivocerebellar dysfunction promotes motor disease phenotypes and identify the cerebellar nuclei as a therapeutic target for surgical intervention.

## Abstract (web summary)

Dystonia is thought to be driven by impairments in cerebellar signalling. The authors use a mouse genetic approach to silence excitatory transmission in the inferior olive to cerebellum pathway, resulting in dystonia-like signs in the animals which can be alleviated using DBS stimulation of the pathway.

Dystonia is an incurable neurological disorder that is defined by abnormal muscle contractions and repetitive twisting of affected body parts. These symptoms intensify during movement^1^. Dystonia can occur either as an independent disease or as a comorbid condition with other movement disorders including ataxia, tremor and Parkinson's disease^2^. The age of onset is variable. Hereditary, primary dystonia is common in young children and teens, whereas focal dystonia often affects adults. It is becoming clear that dystonia is a result of an aberrant motor network, and recent work points to the cerebellum via the basal ganglia as capable of instigating dystonia^3^.

The inferior olive projects its axons exclusively to the cerebellum. Among its targets are direct contacts with the Purkinje cell dendrites via projections called climbing fibres. Climbing fibres induce a unique action potential called the complex spike^4^. The climbing fibre–Purkinje cell synapse mediates the predominant mode of olivocerebellar communication^5^. It coordinates the precise timing of motor commands, although it may also control motor learning and error correction during movement^4^. Climbing fibres are excitatory; they release glutamate and modulate Purkinje cell activity. Accordingly, *in vivo*, the complex spike action potentials are powerful enough to control the intrinsic firing of Purkinje cells^6^ that control the pattern of firing in the cerebellar nuclei^7^,^8^.

In specific cases of human dystonia, the inferior olive was found to have metabolic defects and it was infiltrated by microglia and macrophages^9^,^10^. In rodent models, electrophysiological analyses revealed defects in cerebellar activity and olive communication^11^. In particular, in the dystonic rat *dt*, complex spike firing rate is lower than normal and the Purkinje cells and cerebellar nuclei exhibit abnormal burst firing^12^. Previous work using lesioning methods to destroy the inferior olive also suggested its potential for initiating dystonic postures^13^.

To further address the role of the inferior olive in dystonia, here we sought to manipulate olivocerebellar activity, and then test whether motor behaviours that resemble dystonia arise. Our goal was to develop a noninvasive *in vivo* approach for altering olivocerebellar function. We therefore devised a conditional mouse genetic model to test the hypothesis that loss of olivocerebellar function triggers cerebellar defects that cause dystonia-like behaviour. In the model, eliminating excitatory synaptic neurotransmission between the inferior olive and cerebellum selectively targeted olivocerebellar communication. To examine if and how dystonia-like behaviours emerge we combined our genetic approach with behavioural paradigms, *in vivo* electrophysiology, molecular expression analysis and anatomical examination. We also tested whether cerebellar neuromodulation is an effective approach for alleviating dystonia using reversible chemical lesions and deep brain stimulation (DBS). The data uncovered a cerebellar circuit origin of dystonia-like behaviour and its mechanism of action. The data also provided new opportunities for restoring movement with cerebellar directed DBS.

## Results

### A genetic strategy for targeting olivocerebellar activity

**Figure 1:**
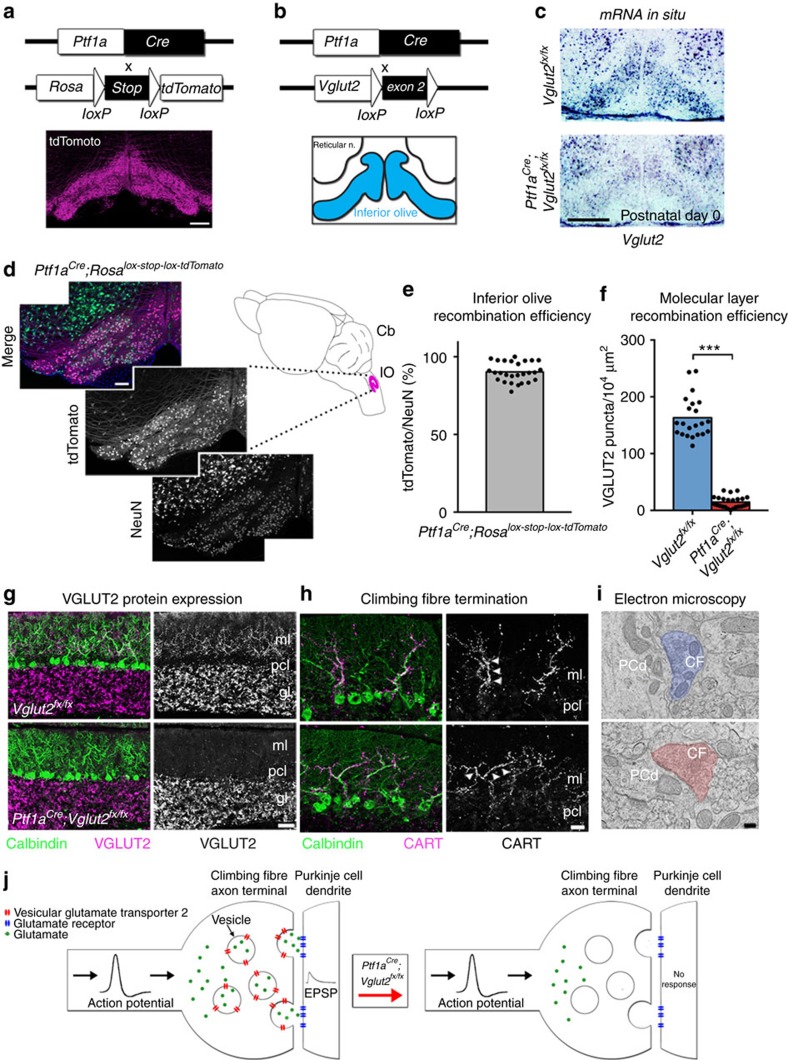
An *in vivo* conditional genetic strategy for silencing neurotransmission at excitatory olivocerebellar axon terminals in mice. (**a**) *Ptf1a*^*Cre*^ can be used to target the inferior olivary nucleus as demonstrated by tdTomato expression. Scale bar, 200 μm. (**b**) Using the same *Ptf1a*^*Cre*^ driver, exon 2 of *Vglut2* can be selectively removed and *Vglut2* deleted from the inferior olive. (**c**) An antisense *in situ* probe for *Vglut2* mRNA reveals its removal from the inferior olive by postnatal day 0. Scale bar, 200 μm. (**d**) *Ptf1a*^*Cre*^-driven tdTomato expression compared with the distribution of NeuN-positive cells in the inferior olive, as schematized in the right panel. Scale bar, 100 μm. Schematic reproduced in part, with permission, from Reeber *et al*.^78^. (**e**) Quantification of the recombination efficiency of *Ptf1a*^*Cre*^ by dividing the number of tdTomato-positive cells by the number of NeuN-positive cells in the inferior olive in *Ptf1a*^*Cre*^*;Rosa*^*lox-stop-lox-tdTomato*^ mice; *n*=3 mice. (**f**) Quantification of VGLUT2 protein expression in the molecular layer of lobules in all four transverse zones of the cerebellum show a significant reduction in *Ptf1a*^*Cre*^*;Vglut2*^*fx/fx*^ mice (*P*=9.294 × 10^−16^; Student's unpaired *t*-test); *n*=4 mice of each genotype. ***P<0.001. (**g**) Examples of expression of VGLUT2 protein in the molecular layer of lobule VI in a *Vglut2*^*fx/fx*^ and a *Ptf1a*^*Cre*^*;Vglut2*^*fx/fx*^ mouse. Scale bar, 50 μm. (**h**) CART expression in climbing fibres in the molecular layer of lobule IX in a *Vglut2*^*fx/fx*^ and a *Ptf1a*^*Cre*^*;Vglut2*^*fx/fx*^ mouse. Note that although CART expression in lobules IX and X is predominant and heaviest in climbing fibres, in other lobules it does label a subset of mossy fibres and scattered beaded fibres^19^. Arrowheads point to climbing fibres. Scale bar, 20 μm. (**i**) Electron microscopy showing the ultrastructure of climbing fibre synapses onto large, Purkinje cell dendritic branches in a *Vglut2*^*fx/fx*^ and a *Ptf1a*^*Cre*^*;Vglut2*^*fx/fx*^ mouse. Scale bar, 200 nm; *n*=4 mice of each genotype. (**j**) Schematic depicting the outcome of genetically silencing climbing fibre terminals. CF, climbing fibre; gl, granular layer; pcl, Purkinje cell layer; ml, molecular layer; PCd, Purkinje cell dendrite; CART, cocaine- and amphetamine-related transcript.

We exploited a unique intersection between the expression patterns of two genes to conditionally block excitatory neurotransmission from the inferior olive to the cerebellum in mice. Specifically, we removed *vesicular glutamate transporter 2* (*Vglut2*) from the olive using a *Ptf1a*^*Cre*^ driver allele (Fig. 1a,b; see Methods). *Ptf1a* encodes a bHLH transcription factor that is primarily expressed in inhibitory neurons in which it controls cell lineage in discrete structures in the brain (hypothalamus and cerebellum^14^; retina^15^; spinal cord^16^), except for in the inferior olive where it controls excitatory neuron development^17^ (Fig. 1c). *Vglut2* encodes a transporter protein required for loading the neurotransmitter glutamate into presynaptic vesicles for chemical synaptic signalling in excitatory cells^18^ and is the vesicular transporter expressed in axon terminals of inferior olivary neurons. We postulated that *Ptf1a* and *Vglut2* expression should intersect in the inferior olive, since the olive is the only glutamatergic brain structure known to express *Ptf1a*^17^. We first crossed the *Ptf1a*^*Cre*^ mice to a *Rosa*^*TdTomato*^ reporter to test the recombination efficiency in the inferior olive (Supplementary Fig. 1). By examining red fluorescent protein-positive cells in combination with NeuN expression in the olive, we found that *Ptf1a*^*Cre*^*;Rosa*^*lox-stop-lox-tdTomato*^ mice exhibited ∼90% recombination (90±3.5%; *n*=27 sections from 3 animals; Fig. 1d,e). Moreover, consistent with previous data, we found limited recombination anterior to the hindbrain^14^ (cerebral cortex and hypothalamus; Supplementary Fig. 1a–i). Next, we crossed the *Ptf1a*^*Cre*^ mice to mice with *floxed* alleles of *Vglut2* (*Vglut2*^*flox*^=*Vglut2*^*fx*^). Based on when the two genes turn on, we anticipated that *Vglut2* would be deleted from inferior olivary cells as they migrate towards the base of the brainstem—thus, climbing fibres should not express VGLUT2 and therefore would be unable to signal with glutamate (Fig. 1j). Analysis of mRNA expression in neonatal (P0) and adult mice using *in situ* hybridization showed that in the *Ptf1a*^*Cre*^*;Vglut2*^*fx/fx*^ mutant mice *Vglut2* was substantially reduced in the olive, but not in the surrounding brainstem nuclei that project mossy fibres to the cerebellum (Fig. 1c). Accordingly, in the cerebellar cortex, VGLUT2 protein was eliminated from the molecular layer, the site of termination of climbing fibres (Fig. 1g,h), with an efficiency of ∼91% across the cerebellum (Fig. 1f and Supplementary Table 1). In contrast, mossy fibre terminals in the granular layer still expressed VGLUT2 with a similar distribution between *Vglut2*^*fx/fx*^ and *Ptf1a*^*Cre*^*;Vglut2*^*fx/fx*^mice (Fig. 1g and Supplementary Table 1). CART, which is heavily expressed in a subset of climbing fibres in lobules IX and X,^19^ and transmission electron microscopy revealed that deleting *Vglut2* in the inferior olive did not disrupt the termination of climbing fibres in the molecular layer or their targeting onto the shafts of Purkinje cell dendrites (Fig. 1h,i).

**Figure 2:**
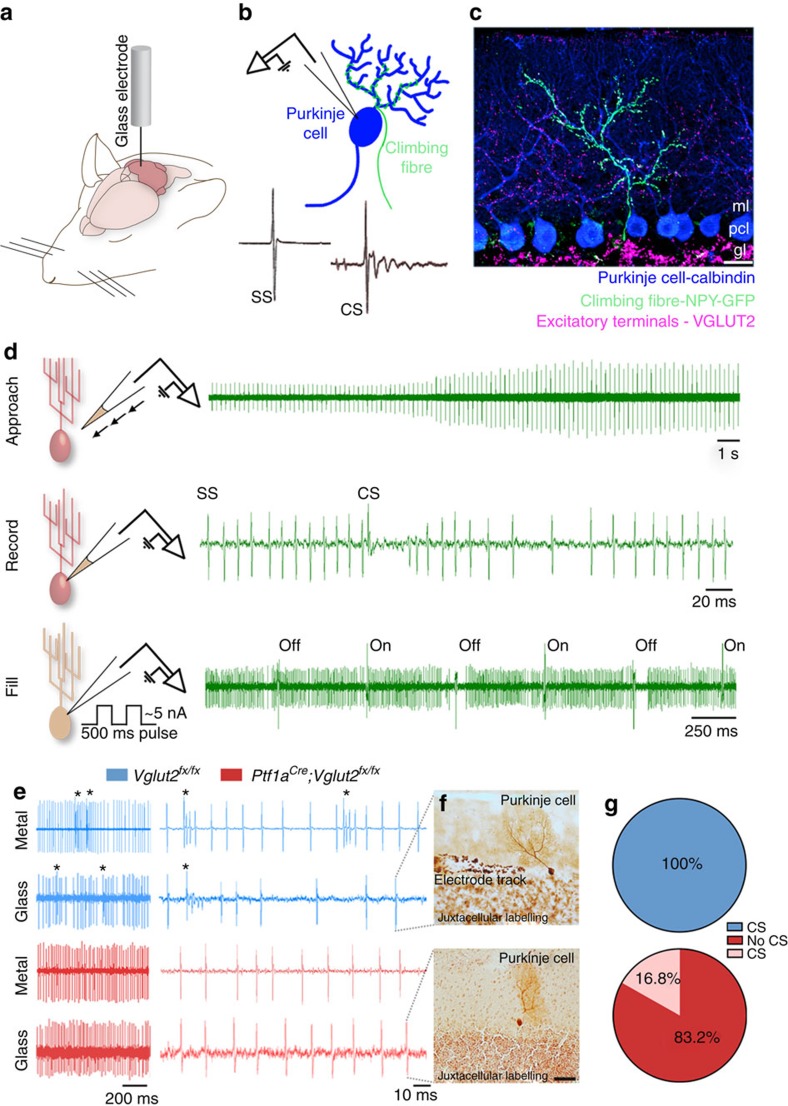
Loss of VGLUT2 eliminates climbing fibre-driven complex spikes. (**a**) A schematic showing a cerebellar recording in an anaesthetized mouse. Schematic adapted with permission, from drawings published in ref. 28. (**b**) A schematic depicting a single-unit recording from a Purkinje cell with its climbing fibre. Samples of a simple spike (SS) and a climbing fibre-driven complex spike (CS) are shown. (**c**) Molecular expression showing the one-to-one relationship between a climbing fibre and a Purkinje cell with VGLUT2 marking the climbing fibre terminals. Scale bar, 20 μm. (**d**) A schematic of the juxtacellular labelling process. Current pulses are used to find cells based on increased resistance. Once a cell is found, the recording step begins by loosely patching on the membrane of the cell, and recording its extracellular spiking activity. After recording for a sufficient period of time, the cell is filled by delivering neurobiotin using 500 ms pulses of positive current between 1 and 5 nA for ∼1 h. Schematic adapted with permission, from drawings published in ref. 74. (**e**) Examples of traces from 3-month-old adult *Vglut2*^*fx/fx*^ and *Ptf1a*^*Cre*^*;Vglut2*^*fx/fx*^ Purkinje cells either using metal electrodes for extracellular recording or glass electrodes for juxtacellular recording. Complex spikes are labelled with asterisks. Note the lack of complex spikes in the mutant traces. (**f**) Examples of filled Purkinje cells in adult 3–5-month-old *Vglut2*^*fx/fx*^ and *Ptf1a*^*Cre*^*;Vglut2*^*fx/fx*^ mice. *n*>4 cells for each genotype. Scale bar, 50 μm. (**g**) Quantification of the number of Purkinje cells with identifiable complex spikes in *Vglut2*^*fx/fx*^ mice compared with recordings from *Ptf1a*^*Cre*^*;Vglut2*^*fx/fx*^ mice of all ages from postnatal day 19 to 8 months. This analysis includes data from all of our electrophysiology experiments. The Purkinje cells were recorded from all lobules of the vermis and from crusI and II of the hemispheres. gl, granular layer; pcl, Purkinje cell layer; ml, molecular layer; NPY, neuropeptide Y (in this case a transgenic allele drives GFP expression).

Loss of VGLUT2 in climbing fibres should eliminate Purkinje cell complex spike activity. We tested this using extracellular, *in vivo* electrophysiology recordings (Fig. 2a–c). The recordings revealed the cellular-level precision of the genetic approach as complex spikes were absent in most Purkinje cells whereas simple spikes persisted (Fig. 2e). We then used a loose-patch, juxtacellular recording approach in which we used glass electrodes to fill a subset of Purkinje cells with neurobiotin after recording to confirm cellular identity after isolating single units *in vivo* (Fig. 2d–f). From a total of 119 recorded neurons in *Ptf1a*^*Cre*^*;Vglut2*^*fx/fx*^ mice, we only found 20 mutant Purkinje cells with complex spikes (16.8%; Fig. 2g). However, the histological confirmation of recorded Purkinje cells was not possible in the metal electrode recordings (Fig. 2e). We therefore limited the potential for erroneous classification of neurons first using stereotaxic coordinates; cerebellar nuclear neurons are located deep within the core of control and mutant cerebella and therefore should not be easily confused with more superficially recorded Purkinje cells. Second, we assessed each cell for spike waveform; Purkinje cell simple spikes, which were preserved in the mutants, had a distinct waveform. Still, even with these reliable criteria we cannot guarantee the correct identification of every recorded cell. Severing the interaction between climbing fibres and Purkinje cells did not cause regression of climbing fibres, cell loss, changes in cell distribution (Supplementary Fig. 2a–n) or a compensatory increase in *Vglut1* mRNA in the inferior olive or VGLUT1 protein in the molecular layer as measured by mean pixel intensity in fluorescence images (Supplementary Fig. 3a–c; Supplementary Table 1). The data showed that *Ptf1a*^*Cre*^*;Vglut2*^*fx/fx*^ mice had anatomically intact Purkinje cell microcircuits with signalling from the inferior olive to cerebellar cortex obstructed by a deficit of complex spikes in all lobules.

### *Ptf1a*^*Cre*^*;Vglut2*^*fx/fx*^mice have dystonia-like behaviours

**Figure 3:**
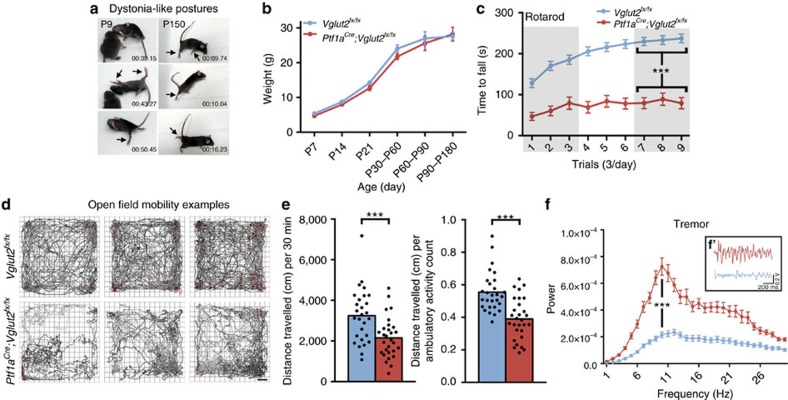
Blocking climbing fibre function causes severe dystonia-like behaviours. (**a**) Serial stills of both early postnatal (with a control *Vglut2*^*fx/fx*^ littermate shown) and adult *Ptf1a*^*Cre*^*;Vglut2*^*fx/fx*^mice displaying dystonia-like postures. Mobility is severely impaired and their limbs are extended and stiff. Arrows identify extended, stiff limbs. (**b**) Weights of mice at different ages (postnatal day (P)7, P14, P21, P30–P60, P60–P90 and P90–P180) reveal no significant differences between control and mutant mice (at least 8 mice from each genotype for each age, gender-matched, Student's unpaired *t*-test). Note that although movement is abnormal in the mutants, they are still capable of eating, exploring the cage and mating. Error bars are defined as s.e.m. (**c**) Rotarod performance is severely impaired in the mutant mice; *n*=62 *Vglut2*^*fx/fx*^ mice, 38 *Ptf1a*^*Cre*^*;Vglut2*^*fx/fx*^ mice. Performance of the last day is significantly different between control and mutant mice (*P*=1.746 × 10^−14^; Student's unpaired *t*-test). Error bars are defined as s.e.m. (**d**) Examples of open field activity from three different *Vglut2*^*fx/fx*^ controls and three different *Ptf1a*^*Cre*^*;Vglut2*^*fx/fx*^ mutant mice showing the range of performance for both genotypes. Scale bar, 5 cm. (**e**) Quantification of *Vglut2*^*fx/fx*^ (*n*=28 mice) and *Ptf1a*^*Cre*^*;Vglut2*^*fx/fx*^ (*n*=29) open field mobility over a 30 min period. The left graph shows a decrease in the total distance travelled in the mutants (*P*=9.62 × 10^−4^; Student's unpaired *t*-test) and the right graph shows a lower total distance travelled divided by the number of ambulatory events in the mutant mice (*P*=7.20 × 10^−6^; Student's unpaired *t*-test). Although the mutant mice exhibit periods of twisting with limited movement and sustained muscle contractions (see EMG in Supplementary Fig. 4d), they are typically capable of moving around albeit with impaired motion and altered posture (Supplementary Movie 3). (**f**) Tremor is significantly increased in *Ptf1a*^*Cre*^*;Vglut2*^*fx/fx*^ mutant mice. Raw waveforms are passed through a fast Fourier transform and the results graphed as power versus frequency; *n*=139 *Vglut2*^*fx/fx*^ and 129 *Ptf1a*^*Cre*^*;Vglut2*^*fx/fx*^ mice. Tremor in all mice was of similar frequency (*P*=0.705; Student's unpaired *t*-test). Amplitude is significantly higher in *Ptf1a*^*Cre*^*;Vglut2*^*fx/fx*^ mice (*P*=1.05 × 10^−6^; Student's unpaired *t*-test). (**f**′) Raw tremor waveforms from *Vglut2*^*fx/fx*^ and *Ptf1a*^*Cre*^*;Vglut2*^*fx/fx*^mice. Error bars are defined as s.e.m. ***P<0.001.

Dystonia-like hyperextension of the limbs and twisting of the torso was obvious in perinatal *Ptf1a*^*Cre*^*;Vglut2*^*fx/fx*^ mice with motor phenotypes clearly observed in the mutants by postnatal day (P) 7 (Fig. 3a and Supplementary Movie 1). Mature mice exhibited overt dystonic postures (Supplementary Movies 2 and 3). We used a dystonia rating scale (see Methods) to quantify the behaviour and found that the mutants scored in the severe range. No significant body weight differences were found at any age measured (Fig. 3b). Performance on rotarod was impaired in *Ptf1a*^*Cre*^*;Vglut2*^*fx/fx*^ mice (Fig. 3c and Supplementary Table 1). Open field analysis over 30 min showed that *Ptf1a*^*Cre*^*;Vglut2*^*fx/fx*^ mice were significantly less mobile compared with *Vglut2*^*fx/fx*^ mice (Fig. 3d,e and Supplementary Table 1). Notably, although *Ptf1a*^*Cre*^*;Vglut2*^*fx/fx*^ mice exhibited dystonic postures, they were often able to propel themselves albeit in a poorly coordinated way (Supplementary Movies 2 and 3). Dystonia-like behaviour in *Ptf1a*^*Cre*^*;Vglut2*^*fx/fx*^ mice was distinct from the ‘dystonic attacks' observed in *tottering* mice^20^ because the periods of twisting with sustained muscle contractions (electromyography (EMG); Supplementary Fig. 4d) were not induced or predictable, and although the dystonic postures were not continuous or always obvious, the abnormal locomotion and impaired mobility were chronic. The sustained and sometimes repetitive muscle contractions in dystonia not only restrict normal movement, but often cause tremulous activity^1^,^21^. We therefore used a tremor monitor to test whether the twisting and hyperextension in the *Ptf1a*^*Cre*^*;Vglut2*^*fx/fx*^ mice translated into a particular frequency and power of rhythmic, shaking movements. Typically, the peak of cerebellar-related tremors occurs at frequencies between 4 and 14 Hz. The peaks of ‘physiological tremor' in *Vglut2*^*fx/fx*^ and the tremor in *Ptf1a*^*Cre*^*;Vglut2*^*fx/fx*^ mice both occurred between 4 and 14 Hz (Fig. 3f and Supplementary Table 1). *Ptf1a*^*Cre*^*;Vglut2*^*fx/fx*^ mice had a higher power (Fig. 3f and Supplementary Table 1). However, there are other pathological signals that can alter ongoing motor behaviour. For example, seizures can lead to abnormal movements that could cause or contribute to the dystonia-like behaviour. Importantly, there is a critical link between cerebellar output and seizure activity^22^,^23^. To test whether the behavioural phenotype of the *Ptf1a*^*Cre*^*;Vglut2*^*fx/fx*^ mice was due to seizure activity, we compared electrocorticography (ECoG) recordings between control, dystonic and control mice with kainate-induced seizures (Supplementary Fig. 4a–d). The recordings suggested that *Ptf1a*^*Cre*^*;Vglut2*^*fx/fx*^ mice did not have seizures (Supplementary Fig. 4b–d, Supplementary Table 1 and Supplementary Movies 4–6).

### Deletion of *Vglut2* alters Purkinje cell firing *in vivo*

**Figure 4:**
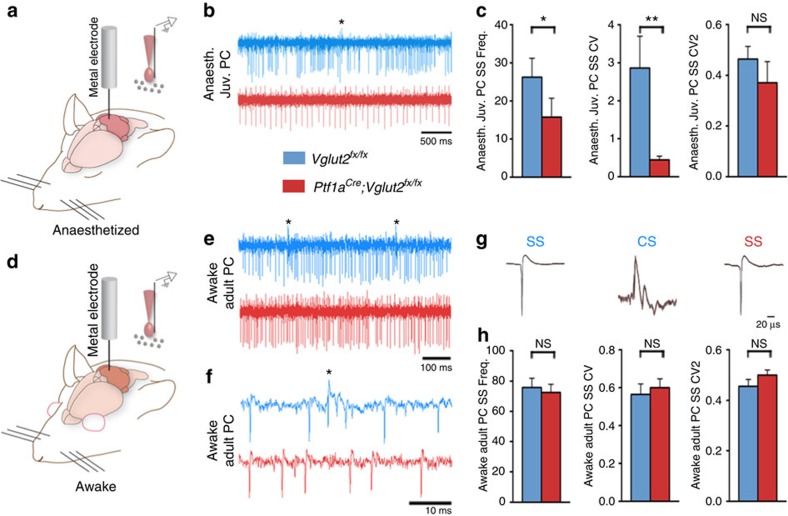
Purkinje cell simple spike firing is abnormal in postnatal developing *Ptf1a*^*Cre*^*;Vglut2*^*fx/fx*^ mutants but normalizes by adulthood. (**a**) A schematized image of the experimental procedure for extracellular recording of single units in an anaesthetized (Anaesth.) mouse and a schematized image of the electrode placement near a Purkinje cell. Schematic adapted with permission, from drawings published in refs 28, 74. (**b**) Low power traces illustrating the firing properties of postnatal Purkinje cells (PC) (ages P19–P21). A complex spike is labelled with an asterisk. Scale bar 500 ms. (**c**) The P19–P21 *Ptf1a*^*Cre*^*;Vglut2*^*fx/fx*^ Purkinje cells (12 cells from 3 mice) fire slowly (frequency; *P*=4.1 × 10^−2^; Student's unpaired *t*-test), with more overall regularity (CV, *P*=3.7 × 10^−3^; Student's unpaired *t*-test) as compared with *Vglut2*^*fx/fx*^ cells (7 cells from 2 mice), but with no significant difference in local regularity (CV2, *P*=0.593; Student's unpaired *t*-test). Error bars are defined as s.e.m. *P<0.05 and **P<0.01. (**d**) A schematized image of the experimental procedure in an awake mouse and a schematized image of the electrode placement near a Purkinje cell. (**e**) Low power traces of awake Purkinje cell recordings in adult 5–8-month-old *Vglut2*^*fx/fx*^ and *Ptf1a*^*Cre*^*;Vglut2*^*fx/fx*^ mice. Complex spikes are labelled with asterisks. Scale bar, 100 ms. (**f**) High power traces of awake Purkinje cell recordings in adult 5–8-month-old *Vglut2*^*fx/fx*^ and *Ptf1a*^*Cre*^*;Vglut2*^*fx/fx*^ mice. A complex spike is labelled with an asterisk. Scale bar 10 ms. (**g**) Sample waveform averages of simple spikes (SS) from adult 5–8-month-old *Vglut2*^*fx/fx*^ and *Ptf1a*^*Cre*^*;Vglut2*^*fx/fx*^ mice and complex spikes (CS) from *Vglut2*^*fx/fx*^ mice. (**h**) Simple spike firing was unchanged in terms of frequency, CV and CV2 in awake, adult *Ptf1a*^*Cre*^*;Vglut2*^*fx/fx*^ mice as compared with *Vglut2*^*fx/fx*^ mice (respectively: *P*=0.695; *P*=0.622; *P*=0.204; ages 5–8 months; *n*=18 *Vglut2*^*fx/fx*^ cells from 7 mice and 17 *Ptf1a*^*Cre*^*;Vglut2*^*fx/fx*^ cells from 11 mice; Student's unpaired *t*-test). Error bars are defined as s.e.m.

To understand how these motor deficits arose, we examined the electrophysiological properties of Purkinje cells, interneurons and cerebellar nuclei neurons because abnormal cerebellar function is implicated in dystonia^12^,^24^. We recorded Purkinje cell firing in anaesthetized mice to examine spontaneous activity in P19–P21 juvenile mice at weaning age (Fig. 4a). We measured their frequency (spikes s^−1^; Hz), coefficient of variance (CV; overall regularity) and CV2 (local regularity). Compared with control littermates, frequency (Fig. 4b,c) and CV (Fig. 4b,c) were significantly lower in the mutants, although CV2 was not changed (Fig. 4b,c). At 5 months of age, when the mice reached adulthood, Purkinje cell firing normalized in the mutants and was no longer abnormal with the exception of the absence of complex spikes (Supplementary Table 1). It is interesting that Purkinje cell function also recovers after the olive is lesioned with 3-acetylpyridine^25^. Purkinje cells are not the only cerebellar cortical neurons to receive input from climbing fibres. Climbing fibres signal to molecular layer interneurons by glutamate spillover^26^. Loss of VGLUT2 in climbing fibres could therefore alter interneuron firing. We found similar firing properties in putative stellate cell and basket cell interneurons in *Ptf1a*^*Cre*^*;Vglut2*^*fx/fx*^ compared with *Vglut2*^*fx/fx*^ mice (Supplementary Fig. 5a–c).

We next used an awake recording approach in head-fixed, behaving mice to examine how Purkinje cells in the *Ptf1a*^*Cre*^*;Vglut2*^*fx/fx*^ mice fired during active behaviour (Fig. 4d–h). Similar to what we observed in the anaesthetized adult mice, we found no difference between awake, adult *Vglut2*^*fx/fx*^ and *Ptf1a*^*Cre*^*;Vglut2*^*fx/fx*^ Purkinje cells (Fig. 4e–h and Supplementary Table 1).

**Figure 5:**
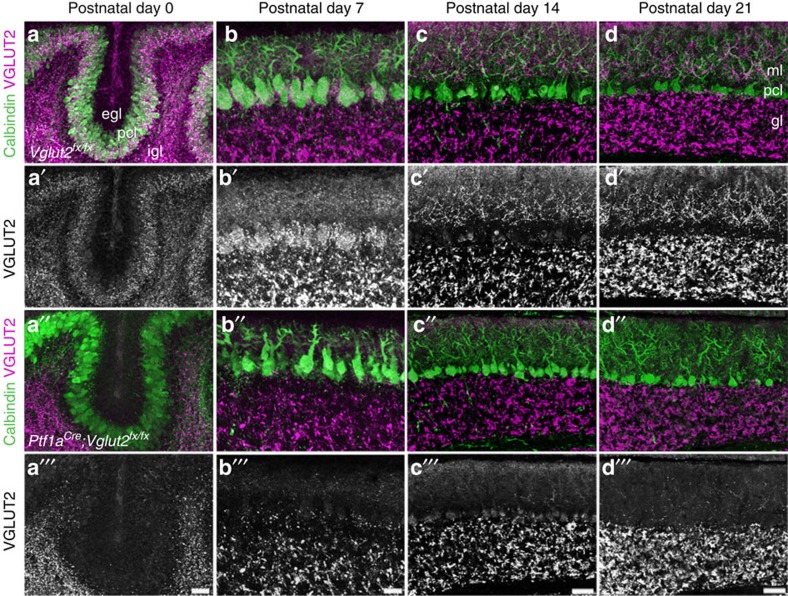
Molecular layer thickness is abnormal in *Ptf1a*^*Cre*^*;Vglut2*^*fx/fx*^ mutant cerebella during the early postnatal ages but recovers with age. (**a**) At postnatal day 0, the Purkinje cell layer is thinner in *Ptf1a*^*Cre*^*;Vglut2*^*fx/fx*^ mutants and VGLUT2 expression is not present in the Purkinje cell layer. (**b**) At postnatal day 7, the molecular layer is significantly thinner in the mutants and there is little to no VGLUT2 expression in the Purkinje cell layer (*P*=2.3 × 10^−4^). (**c**) At postnatal day 14, the Purkinje cell layer in *Ptf1a*^*Cre*^*;Vglut2*^*fx/fx*^ mutants is only slightly thinner than the molecular layer of *Vglut2*^*fx/fx*^ controls and VGLUT2 expression is largely absent in the Purkinje cell layer. (**d**) At postnatal day 21, the Purkinje cell layer in *Vglut2*^*fx/fx*^ mice is nearly the same thickness as that observed in *Ptf1a*^*Cre*^*;Vglut2*^*fx/fx*^ mice even though VGLUT2 expression is eliminated from the Purkinje cell layer. egl, external granular layer; pcl, Purkinje cell layer; igl, internal granular layer; ml, molecular layer; gl, granular layer. Scale bars (**a**''' and **b**'''), 20 μm. Scale bars (**c**''' and **d**'''), 50 μm.

Structural abnormalities are often subtle in dystonia. However, the length of the primary Purkinje cell dendrite is reduced in a knock-in mouse model of *Dyt1* (ref. 27). To test whether there are similar defects in the *Ptf1a*^*Cre*^*;Vglut2*^*fx/fx*^ mice, we measured molecular layer thickness as a proxy for dendrite span. Molecular layer thickness is a sensitive and straightforward measure for developmental and disease-associated defects that disrupt Purkinje cell dendrite size and/or the placement of Purkinje cells into a perfect monolayer^28^. At birth and during postnatal development, molecular layer thickness was significantly less in *Ptf1a*^*Cre*^*;Vglut2*^*fx/fx*^ compared with *Vglut2*^*fx/fx*^mice (Fig. 5a–d and Supplementary Table 1). However, by adulthood there was no difference between the genotypes (Supplementary Table 1). We next checked for ectopic upregulation of tyrosine hydroxylase (TH) in *Ptf1a*^*Cre*^*;Vglut2*^*fx/fx*^ Purkinje cells (Supplementary Fig. 6). TH is a precursor for dopamine, norepinephrine and epinephrine. Cerebellar dysfunction is often associated with ectopic upregulation of TH in Purkinje cells; its presence is thought to result from changes in excitability and/or Ca^2+^ dysregulation^11^. However, we did not find an ectopic upregulation of TH in the *Ptf1a*^*Cre*^*;Vglut2*^*fx/fx*^ mice (*n*=4 animals per genotype; Supplementary Fig. 6a–d), despite the presence of dystonia.

### *Vglut2* mutants have irregular firing in cerebellar nuclei

**Figure 6:**
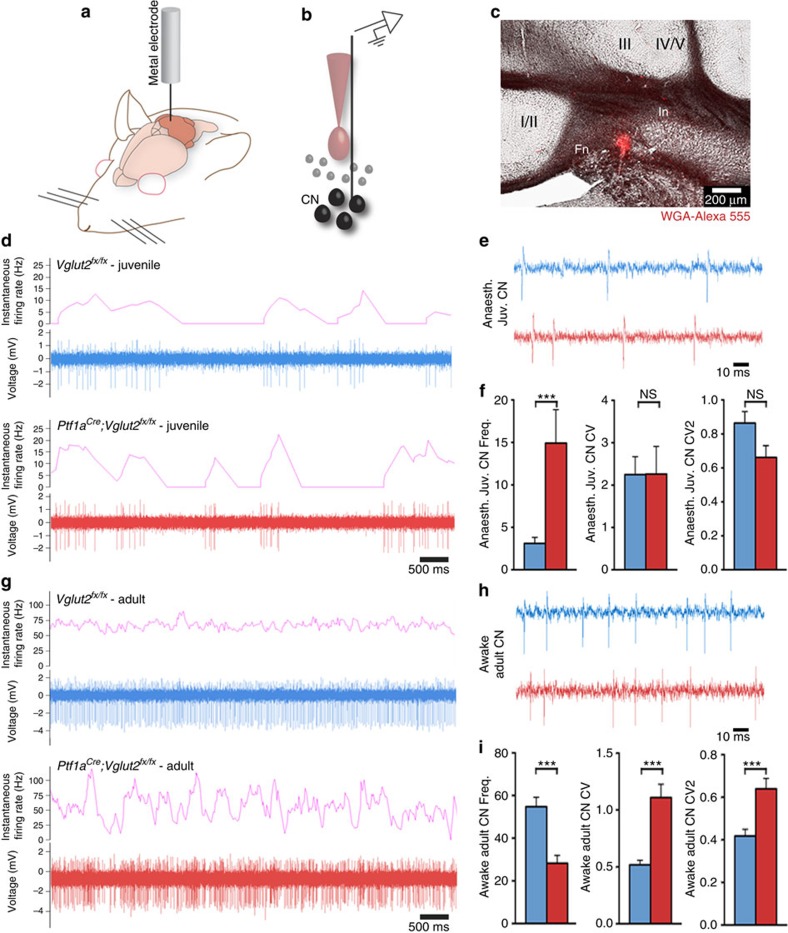
Cerebellar output function is abnormal in dystonic *Ptf1a*^*Cre*^*;Vglut2*^*fx/fx*^ mice. (**a**) A schematic example of a single-unit extracellular recording in the cerebellum of an awake mouse. (**b**) A schematic example of the electrode position relative to the neurons of the cerebellar nuclei (CN). (**c**) WGA-Alexa 555 injection marking the interposed cerebellar nucleus (In). In this example the medial portion of the In was recorded, although in other recordings more lateral In cells were also examined. The fastigial nucleus (Fn), which is the most medially located cerebellar nucleus, is also shown. The surrounding vermis lobules are indicated by Roman numerals. Scale bar 200 μm. (**d**) Low power examples of P19–P21 juvenile *Vglut2*^*fx/fx*^ and *Ptf1a*^*Cre*^*;Vglut2*^*fx/fx*^ cerebellar nuclei neural firing patterns by binning instantaneous firing rate at 400 ms. (**e**) High power traces from neurons in the cerebellar nuclei of juvenile *Vglut2*^*fx/fx*^ and *Ptf1a*^*Cre*^*;Vglut2*^*fx/fx*^ mice. Scale bar, 10 ms. (**f**) Quantifications of juvenile cerebellar nuclear neuron frequency (*P*=1.35 × 10^−2^; Student's unpaired *t*-test), CV (*P*=0.98; Student's unpaired *t*-test) and CV2 (*P*=0.053; Student's unpaired *t*-test); *n*=8 *Vglut2*^*fx/fx*^ cells (from 3 mice), 11 *Ptf1a*^*Cre*^*;Vglut2*^*fx/fx*^ cells (from 3 mice). Error bars are defined as s.e.m. ***P<0.001. (**g**) Low power examples of adult *Vglut2*^*fx/fx*^ and *Ptf1a*^*Cre*^*;Vglut2*^*fx/fx*^ cerebellar nuclei neural firing patterns showing instantaneous firing rate binned at 400 ms. (**h**) High power traces of neurons of the cerebellar nuclei in adult *Vglut2*^*fx/fx*^ and *Ptf1a*^*Cre*^*;Vglut2*^*fx/fx*^ mice. Scale bar, 10 ms. (**i**) Quantifications of adult cerebellar nuclear neuron frequency (*P*=3.594 × 10^−5^; Student's unpaired *t*-test), CV (*P*=5.6 × 10^−5^; Student's unpaired *t*-test) and CV2 (*P*=6.56 × 10^−4^; Student's unpaired *t*-test); *n*=33 *Vglut2*^*fx/fx*^ cells (from 14 mice), 27 *Ptf1a*^*Cre*^*;Vglut2*^*fx/fx*^ cells (from 10 mice). Error bars are defined as s.e.m.

Why do *Ptf1a*^*Cre*^*;Vglut2*^*fx/fx*^ mice remain dystonic after Purkinje cell structure and simple spike firing are corrected? Purkinje cells project to three pairs of cerebellar nuclei: the fastigial nuclei (medial), interposed nuclei (middle) and dentate nuclei (lateral). Cerebellar nuclear neurons exhibit abnormal irregular firing in several models of dystonia (dystonic rat^12^, *tottering*^29^, rapid-onset dystonia-parkinsonism^24^ and mutation of *Lamb1* in mouse^30^). These previous data indicate that regardless of how the dystonia is caused, one common consequence might be irregular activity in the cerebellar nuclei. To test this hypothesis, we recorded the activity of the cerebellar nuclear neurons in anaesthetized weanling and awake adult *Ptf1a*^*Cre*^*;Vglut2*^*fx/fx*^ mice (Fig. 6a–c). In accordance with the impact on Purkinje cells, blocking olivocerebellar excitatory neurotransmission induced defects in the firing of developing cerebellar nuclear neurons. In P19–21 mice, we found that the cells in the *Ptf1a*^*Cre*^*;Vglut2*^*fx/fx*^ mice fired at a higher rate than *Vglut2*^*fx/fx*^ mice, although there was no difference in the pattern of firing (Fig. 6d–f and Supplementary Table 1). However, in contrast to the recovery of firing we observed in adult Purkinje cell simple spikes, the loss of climbing fibre input induced permanent defects in the frequency and pattern of firing of cerebellar nuclear neurons (cerebellar nuclei firing is also permanently altered after 3-acetylpyridine^25^). *Ptf1a*^*Cre*^*;Vglut2*^*fx/fx*^ cerebellar nuclei had a significantly lower firing frequency, higher CV and higher CV2 (Fig. 6g–i and Supplementary Table 1). The relatively high CV and CV2 indicated that the loss of climbing fibre neurotransmission caused highly irregular cerebellar output at the level of overall firing and spike-to-spike activity. The highly irregular output pattern in adult mutants was reminiscent of the irregular firing in the young mice of both genotypes (Fig. 6d–i), indicating that whereas the *Vglut2*^*fx/fx*^ cells became more regular over time, *Ptf1a*^*Cre*^*;Vglut2*^*fx/fx*^ cells in juvenile mice fired erratically and remained so into adulthood. Analysis of VGAT and VGLUT2 expression revealed that these firing defects were not accompanied by obvious changes in the expression of inhibitory and excitatory presynaptic markers that label terminals in the cerebellar nuclei (Supplementary Fig. 7a–d).

### Abnormal cerebellar nuclei activity drives motor dysfunction

**Figure 7:**
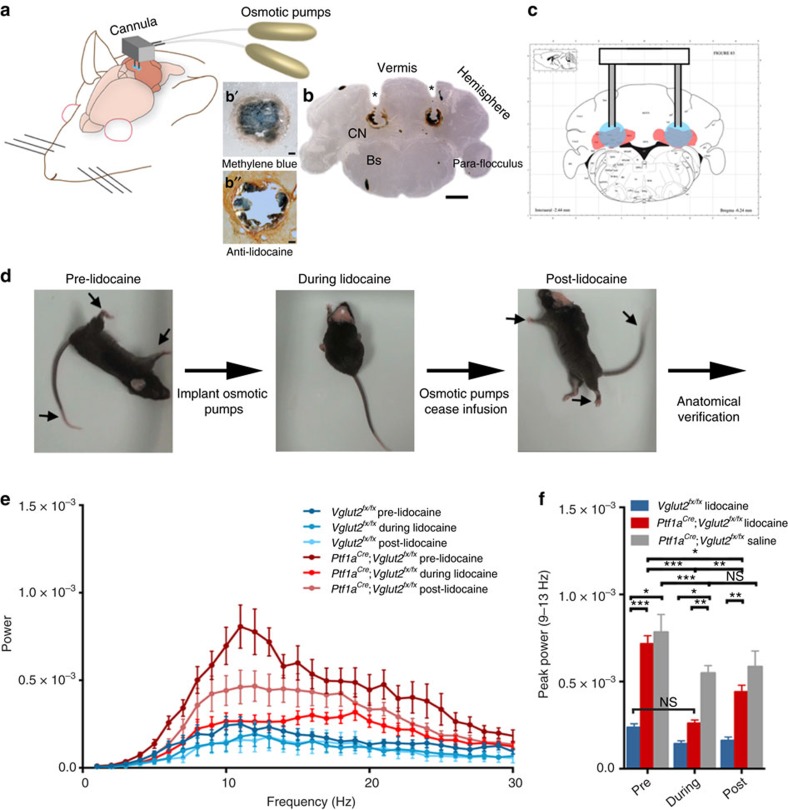
Lidocaine delivery to the interposed cerebellar nuclei eliminates tremor and improves movement in *Ptf1a*^*Cre*^*;Vglut2*^*fx/fx*^ mutant mice. (**a**) A schematic example of delivery of lidocaine via osmotic pumps to a mouse cerebellum. Schematic adapted with permission, from drawings published in ref. 28. (**b**) Anatomical verification of surgical targeting of the cannula to the cerebellar nuclei. In the higher power view of where the cannula tip was located (**b**′), the methylene blue staining approximates the local impact of the lidocaine injection and (**b**”) the lidocaine immunodetection was used to examine the local spread of lidocaine within the cerebellar nuclei. Scale bar in (**b**), 1 mm. Scale bar in (**b**' and **b**”), 100 μm. (**c**) Atlas schematic^75^ demonstrating the location of the cerebellar nuclei (highlighted in red) and the bilateral infusion of lidocaine into the interposed nuclei (highlighted in blue). Schematic reproduced in part, with permission, from Paxinos and Franklin^75^. (**d**) Stills of videos of a dystonic mouse before, during and after lidocaine infusion. Arrows point to stiff extended limbs and tail. (**e**) Tremor power/frequency graph of *Vglut2*^*fx/fx*^ and *Ptf1a*^*Cre*^*;Vglut2*^*fx/fx*^ mice before, during and after lidocaine infusion. Error bars are defined as s.e.m. (**f**) Quantification of peak power of *Vglut2*^*fx/fx*^ (pre versus during: Student's paired *t*-test *P* value=0.1117), *Ptf1a*^*Cre*^*;Vglut2*^*fx/fx*^ (pre versus during: Student's paired *t*-test *P* value=0.0001) and *Ptf1a*^*Cre*^*;Vglut2*^*fx/fx*^ sham (pre versus during: Student's paired *t*-test *P* value=0.0068; during versus post: Student's paired *t*-test *P* value=0.2941). *Vglut2*^*fx/fx*^ during versus *Ptf1a*^*Cre*^*;Vglut2*^*fx/fx*^ during Student's unpaired *t*-test *P* value=0.131; *n*=7 *Vglut2*^*fx/fx*^ mice with lidocaine, 14 *Ptf1a*^*Cre*^*;Vglut2*^*fx/fx*^ mice with lidocaine, 4 *Ptf1a*^*Cre*^*;Vglut2*^*fx/fx*^ mice with saline. Error bars are defined as s.e.m. Either paired or unpaired Student's *t*-tests were used as noted. *P<0.05, **P<0.01 and ***P<0.001.

Removal of the entire cerebellum in the dystonic rat, *dt*, eliminates the motor signs that are characteristic of the disease^12^. Additionally, electrolytic and chemical lesions of the cerebellar nuclei significantly improve motor function in *dt*^12^. These studies imply that having no cerebellum is better than having a poorly functioning cerebellum. We reasoned that if abnormal cerebellar nuclei activity was driving the dystonia-like motor abnormalities in *Ptf1a*^*Cre*^*;Vglut2*^*fx/fx*^ mice, then silencing of the cerebellar nuclei output should improve motor function. To reversibly silence the cerebellar nuclei, action potential generation was blocked by infusing the voltage-gated sodium channel blocker lidocaine into the cerebellar nuclei of adult dystonic *Ptf1a*^*Cre*^*;Vglut2*^*fx/fx*^ mice. Lidocaine effectively blocks cerebellar firing *in vivo* (see Discussion and Methods). We used tremor as a quantitative measure of abnormal behaviour because of its specific relevance to dystonia^21^. Tremor was measured before, during and after lidocaine infusion using osmotic pumps (Fig. 7a–d). The cannula of the pump was set to deliver 4% lidocaine bilaterally to the centre of the cerebellar nuclei (Fig. 7b,c). We measured the spread of lidocaine indirectly by examining methylene blue that was included in the infusion solution (Fig. 7b′) or directly by using an anti-lidocaine antibody for immunodetection (Fig. 7b′′). The lidocaine remained mainly localized to the intended target, the interposed nuclei, with only limited spread into the adjacent fastigial and dentate nuclei. Dystonic tremor in *Ptf1a*^*Cre*^*;Vglut2*^*fx/fx*^ mice was significantly reduced upon lidocaine infusion (Fig. 7e,f). The overall movement of the lidocaine-treated mutant mice improved (Fig. 7d and Supplementary Movie 7). After the pump was depleted of lidocaine, tremor in the mutants returned to a level significantly different from that during lidocaine level (Fig. 7e,f). It is intriguing that infusion of saline into the mutants had a significant effect on tremor (Fig. 7f). However, at the end of saline infusion the tremor did not rise above the during treatment power, which is in contrast to the lidocaine condition, suggesting the basis for tremor reduction with saline infusion is the cannula lesion. Interestingly, lidocaine infusion lowered the physiological tremor in *Vglut2*^*fx/fx*^ mice, although the effect was not statistically significant (Fig. 7f). These findings suggested that the main neural deficit driving the dystonia-like motor abnormalities in the *Ptf1a*^*Cre*^*;Vglut2*^*fx/fx*^ mice was erratic cerebellar nuclear firing. Furthermore, these data raised the possibility that modulating the abnormal output activity of the cerebellar nuclei could have therapeutic benefits.

### Cerebellar DBS recovers mobility

**Figure 8:**
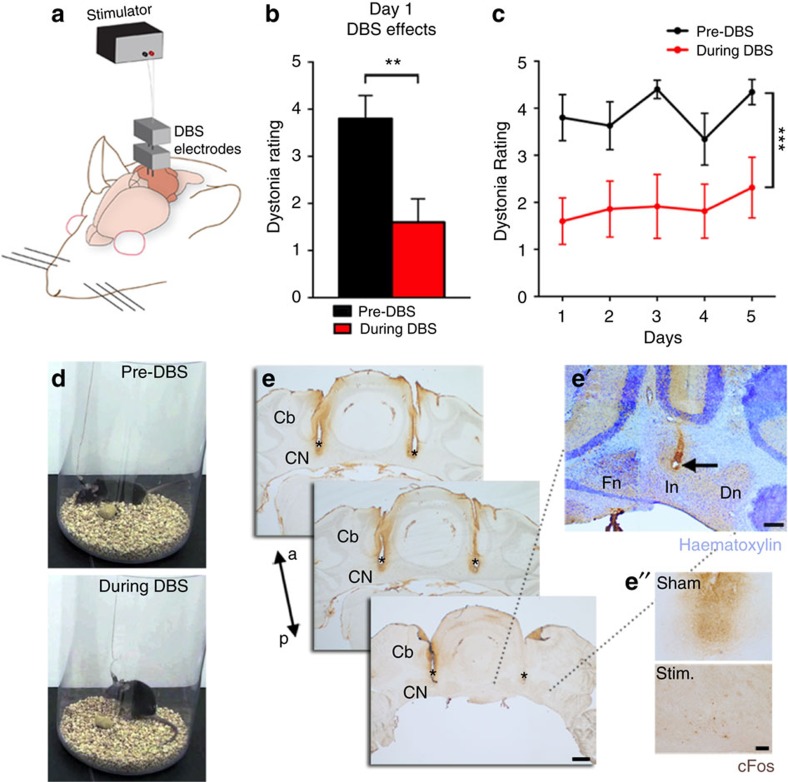
Deep brain stimulation of the interposed cerebellar nuclei restores mobility in severely dystonic mice. (**a**) A schematic example of DBS targeting into the cerebellum. (**b**) Dystonia rating before and during DBS stimulation of the interposed cerebellar nucleus in naive *Ptf1a*^*Cre*^*;Vglut2*^*fx/fx*^ mutant mice (*P*=0.0014; *n*=7 mice; Student's paired *t*-test). There is no significant difference between pre-DBS and post-DBS suggesting limited residual effects once the DBS is turned off (day 1 pre-DBS (3.8±0.49) versus post-DBS (3.075±0.63) Student's paired *t*-test *P* value=0.1099). Error bars are defined as s.e.m. (**c**) Comparison of pre-DBS and during DBS dystonia ratings for each of the 5 days shows no significant difference between days in the pre-DBS dystonic rating or during DBS dystonic measures (pre-DBS days 1–5: F(2.454, 14.72)=2.762; *P*=0.0875; during DBS days 1–5: F(2.647, 15.88)=1.380; *P*=0.2841). A one-way ANOVA shows a between-treatment difference based on averages of pre-DBS, during DBS and post-DBS over 5 days of stimulation: F(2, 12)=31.42 (*P*<0.0001) (see also Supplementary Movie 8 for pre-, during and post-DBS effects). Control mice were not affected by DBS (please see Supplementary Movie 8, *n*=8 *Vglut2*^*fx/fx*^ stimulated and 4 *Ptf1a*^*Cre*^*;Vglut2*^*fx/fx*^ shams). Error bars are defined as s.e.m. **P<0.01 and ***P<0.001. (**d**) Stills of a *Ptf1a*^*Cre*^*;Vglut2*^*fx/fx*^ mouse before and during stimulation. (**e**) Anatomical verification of surgical targeting of the DBS electrodes to the interposed cerebellar nuclei. Scale bar, 500 μm. (**e**') Haematoxylin counterstain (deep blue/purple) showing the site of electrode in the cerebellar nuclei. Scale bar, 200 μm. (**e**'') Compared with the sham, cFos expression (brown) is not upregulated by stimulation. Scale bar, 100 μm. Cb, cerebellum; a, anterior; p, posterior; CN, cerebellar nuclei; Fn, fastigial nucleus; In, interposed nucleus; Dn, dentate nucleus; asterisks, electrode tracks.

DBS is a promising treatment for a number of motor diseases. There is particularly high optimism for its use in dystonia^31^. However, the use of DBS is restricted to targeting mainly the globus pallidus, thalamus and subthalamic nucleus. Based on data in the previous sections, we reasoned that directing DBS to the cerebellar nuclei would be expected to improve motor function. The general concept of DBS is that high-frequency stimulation modulates erroneous neural activity and entrains it to a pattern that normalizes behaviour. Although the exact mechanism(s) of DBS action is unclear^32^, one perspective is that the pulses produce inhibitory neuronal effects on somata that are proximal to the location of the electrode. The inhibitory action is thought to be the direct result of depolarization block through a mechanism involving sodium channel inactivation and potassium current potentiation. However, DBS might also increase and regularize the output of the stimulated region by activating local axons. At the network level, the end result is that the entrainment overrides pathological oscillatory activity. We postulated that the irregular firing of cerebellar nuclear neurons in the *Ptf1a*^*Cre*^*;Vglut2*^*fx/fx*^ mice might be an ideal target for restoring movement with cerebellar DBS. To test this possibility, we implanted DBS electrodes bilaterally into adult dystonic *Ptf1a*^*Cre*^*;Vglut2*^*fx/fx*^ mice and littermate controls (Fig. 8a,d,e,e′). We targeted the interposed nuclei because they project extensively to regions that control ongoing movement, such as the red nucleus^33^. The interposed also projects to several regions of the thalamus^34^ that project to the rest of the motor circuit. We tested three conditions: control implanted (stimulation), mutant implanted sham (no stimulation) and mutant implanted (stimulation). We used a 130 Hz biphasic stimulus (30 μA, 60 μs duration) that has previously been used to mimic human DBS parameters in mouse models of disease^35^. The response to DBS was immediate. Stimulated mutants demonstrated improved mobility. Using the dystonia rating scale, we found that the mutants receiving DBS showed decreased twisting and limb stiffness (Fig. 8b and Supplementary Table 1). There was no significant difference between pre-DBS and post-DBS suggesting limited residual effects once the DBS was turned off. Moreover, based on repeated measures analysis of variance (ANOVA), there was no significant difference between days in the pre-DBS dystonia rating or during DBS measures (Fig. 8c). A one-way ANOVA showed a between-treatment difference based on averages of pre-DBS, during DBS (Fig. 8c) and post-DBS over 5 days of stimulation (Supplementary Movie 8). The treated mutants groomed themselves, explored the enclosure and stood on their hind legs (Supplementary Movie 8). We did not observe cFos upregulation in the interposed cerebellar nuclear neurons near the site of stimulation (Fig. 8e′′), which is in contrast to the high cFos expression observed after pharmacologically activating the nuclei^36^. This implicates an inhibitory effect of DBS on the cerebellar nuclei somata, although it does not rule out a cooperative excitation of axons. The control mice given DBS into the interposed cerebellar nuclei did not develop any noticeable motor dysfunctions at any point during the experiment (Supplementary Movie 8).

The cerebellum and basal ganglia communicate using a disynaptic pathway involving the centrolateral nucleus of the thalamus^37^. This pathway is also responsible for relaying abnormal cerebellar output activity to the basal ganglia to cause dystonia. Given that the centrolateral nuclei are directly innervated by the cerebellar nuclei, we postulated that DBS directed to this thalamic region should also improve mobility (Supplementary Fig. 8a,b). We found that bilateral stimulation of the centrolateral nuclei in *Ptf1a*^*Cre*^*;Vglut2*^*fx/fx*^ mice significantly improved movement (Supplementary Fig. 8c,d and Supplementary Movie 9).

## Discussion

Neurological diseases are difficult to model because, in many cases, their manifestation includes wiring defects, firing abnormalities, plus neurodegeneration. This combination of disease aetiologies also makes it challenging to design effective therapies. To overcome these problems, we devised a mouse genetics approach to selectively silence fast chemical neurotransmission at a single type of connection in the brain—the olivocerebellar synapse. In doing so, we produced a severe motor condition that recapitulated multiple features that are observed in the debilitating forms of human dystonia. The mice exhibited stiff limbs, slow intense twisting, hyperextension of the back, limbs and toes (splayed) and sustained muscle contractions. With this model, we uncovered a circuit mechanism whereby transient defects in the structure and function of Purkinje cells plus an ongoing absence of climbing fibre complex spike firing were associated with permanent, erratic cerebellar nuclei activity, which was the major alteration driving multiple dystonia-like behaviours. We used the mice to find a new DBS target for rescuing motor behaviour.

Climbing fibre activity is thought to be a central requirement for cerebellar functions, ranging from ongoing motor behaviour^38^ to learning^39^,^40^ to error correction^41^. By extrapolation, climbing fibre function could be predicted to have a major impact on motor disease. For these reasons, there have been many attempts to manipulate climbing fibres. Climbing fibres have been experimentally targeted by lesioning the inferior olive with chemicals^42^ or current^43^, cutting the axons in the inferior cerebellar peduncle^44^, electrically activating the fibres^45^, pharmacologically injecting toxins such as harmaline that synchronize inferior olive firing and increase climbing fibre activity^11^, genetically targeting genes that guide axon pathfinding^46^ or innervation^47^, behavioural methods that eliminate the instructive value of the climbing fibre signal^48^ and, more recently, by optogenetics approaches^49^. However, our intersectional genetics approach has the distinct advantage of silencing climbing fibres in all functional regions of the cerebellum, starting during development, and without causing major circuit rearrangements, axon regression or cell death. There is also the added advantage of being able to analyse the long-term effects of silencing an endogenous synaptic mechanism that revealed how the absence of proper climbing fibre excitatory neurotransmission can cause early-onset dystonia-like motor behaviour. The early postnatal movement defects are intriguing given recent work showing conditional deletion of Tor1a. Tor1a is a ubiquitously expressed AAA+ protein residing in the lumen of the endoplasmic reticulum/nuclear envelope space, the impairment of which is linked to the primary dystonia, *DYT1*. Deletion of Tors1a in the mouse brain causes some similar phenotypes to our mice, especially considering a loss of neurons in the cerebellar nuclei^50^. The olive and/or its downstream targets in the cerebellum, red nucleus or thalamus (and basal ganglia) may be central loci that are affected by dystonia mutations. Moreover, it is intriguing that loss of olivary input to the cerebellum resulted in an enhanced level of 10 Hz tremor. These data suggest that olive function normally serves to suppress pathological oscillations by maintaining 10 Hz oscillations at a subthreshold level that has been described extensively *in vitro* using extracellular^51^ as well as patch-clamp recordings^52^ and *in vivo* using whole-cell recordings^53^ and local field potentials^52^.

Loss of climbing fibre complex spike activity in Purkinje cells altered the firing pattern of simple spikes, but this effect was only transient as adult Purkinje cells surprisingly resumed normal simple spike firing (Fig. 4d–h). Our results are consistent with previous work that showed that acute silencing of climbing fibre function, such as cooling the inferior olive^54^ or acute chemical lesion of the inferior olive^55^, alters Purkinje cell firing. However, Purkinje cell simple spike firing returns to normal after long-term lesioning of the inferior olive^25^ and after we silenced olivocerebellar axons. However, one major difference between our results and these previous manipulations is that in the adult mutant mice we did not find a change in simple spike firing, whereas they reported an increase in rate. We suspect that this difference is because our genetic manipulation targets developing Purkinje cells starting during embryogenesis, whereas other manipulations only affect adult Purkinje cells. The source of the difference could also be due to the manner in which simple spikes and complex spikes interact. In the adult, complex spikes are thought to directly suppress simple spikes^6^,^54^. A different relationship probably exists during development, as evidenced by the low firing rate of simple spikes in young mutant Purkinje cells that do not have complex spikes. Several points support this argument. First, the unique trajectories of simple spike and complex spike development indicate some level of independence between the two signals^56^. Second, the firing properties of developing postnatal Purkinje cells are distinct from adults^56^. The loss of complex spikes in young *Ptf1a*^*Cre*^*;Vglut2*^*fx/fx*^ mice would be predicted to substantially drive down Purkinje cell activity—the lower rate of simple spike activity during development increases the contribution of complex spike activity to the overall rate. In addition to the differences in the stages that were manipulated, it is possible that acute drug- and lesion-based approaches cause a fundamentally different cellular response compared to genetic silencing. In addition, our genetic approach specifically targets excitatory neurotransmission, whereas previous methods likely affect multiple (if not all) modes of cell-to-cell signalling.

Regardless of how the inferior olive is manipulated, a common outcome is that Purkinje cell activity recovers to normal, whereas cerebellar nuclear neuron firing remains abnormal^25^ (Fig. 6g–i). There are several potential reasons for the persistence of cerebellar nuclei dysfunction, but first we will consider how the abnormal firing might be initiated. Neural tracing of axons and genetic marking demonstrated that Purkinje cell axons invade the territories of migrating cerebellar nuclear neurons as early as E15.5 in mice^57^. The early loss of glutamatergic signalling from the inferior olive to Purkinje cells in *Ptf1a*^*Cre*^*;Vglut2*^*fx/fx*^ mice raises the interesting possibility that cerebellar nuclear neurons are abnormal starting from before birth. However, the neurofilament heavy (NFH)-positive excitatory cerebellar nuclear neurons in the mutants appear morphologically normal with only modest differences in the molecular profile of afferent termination throughout the nuclei (Supplementary Fig. 7a–d). However, the early deficit of olive input to the cerebellum could alter developmental gene expression and regulation, as well as the intrinsic firing properties in the cerebellar nuclei. Regardless of these changes and despite the recovery of Purkinje cells, complex spike activity is chronically absent in the *Ptf1a*^*Cre*^*;Vglut2*^*fx/fx*^ mice. This is a critical point to consider because complex spikes can directly affect cerebellar nuclear neuron activity^7^. This in turn is important because complex spikes synchronize between Purkinje cells located in narrow bands of Purkinje cells^58^ that converge and terminate within discrete populations of cerebellar nuclear neurons where they might entrain firing^8^,^59^. This precise organization of structure and function is thought to control different aspects of motor behaviour that are consistent with a role for complex spike dysfunction in dystonia^60^. Given the severity of dystonia and underlying cerebellar nuclei defects in the *Ptf1a*^*Cre*^*;Vglut2*^*fx/fx*^ mice, it might be surprising that Purkinje cell function recovers. The cerebellar nuclei not only project out of the cerebellum but they also send inhibitory^61^ and excitatory^62^ feedback projections to the cerebellar cortex, where they synapse on granule cells and Golgi cells to indirectly affect Purkinje cell function. To counter this circuit, however, potentially destructive abnormal feedback from the cerebellar nuclei in *Ptf1a*^*Cre*^*;Vglut2*^*fx/fx*^ mice could be dampened by homeostatic control of the Purkinje cells via their own local cortical feedback to granule cells^63^ or directly to Purkinje cells^64^. Our awake recordings were intended to capture activity during ongoing and quiet behaviour without distinguishing the Purkinje cell dynamics during motion^65^ or learning^62^.

It is interesting that the loss of complex spikes led to a delay in the structural maturation of Purkinje cell dendrites in early postnatal mice. The recovery of the Purkinje cell dendritic span before adulthood indicates a powerful intrinsic capacity for developmental plasticity in Purkinje cells, and it also suggests that afferent input controls the temporal dynamics of Purkinje cell development. Although the proper formation of the Purkinje cell dendrite and the assembly of the cortical microcircuit are required for proper function^57^, how the delay in the *Ptf1a*^*Cre*^*;Vglut2*^*fx/fx*^ mutants affects behaviour is unclear. It is possible that abnormal Purkinje cell structure and the altered firing co-contribute to the early-onset twisting and hyperextension observed at P7, whereas the abnormal circuit firing alone maintains the dystonia throughout adulthood. However, it is important to note that all other aspects of adult cerebellar architecture and connectivity that we examined were also normal after olivocerebellar silencing. Putative inhibitory interneuron firing in the cerebellar cortex was unaltered, further suggesting that olive function plays a precisely timed and likely very specific role in establishing the wiring diagram of the cerebellum. The impact on cerebellar nuclear function was in stark contrast—as discussed above, their firing never recovered. The severe firing pattern abnormalities in the cerebellar nuclei of *Ptf1a*^*Cre*^*;Vglut2*^*fx/fx*^ mice is consistent with a broader role for these cells in different dystonias^3^,^11^,^24^,^30^. If the abnormal cerebellar nuclei output is a major factor driving dystonia-like behaviour, then removing it should reverse these behaviours. To begin addressing this problem, we first asked whether blocking cerebellar nuclei output could improve motor function. We injected lidocaine into the cerebellar nuclei and indeed movement was improved. However, even though it prevents the transmission of neural activity at the soma, one caveat of the approach is that lidocaine can also block activity in axons passing near the nuclei^66^,^67^. However, the impact on incoming axons is minimized in the *Ptf1a*^*Cre*^*;Vglut2*^*fx/fx*^ mice because the olivocerebellar fibres, which carry the most powerful input to the cerebellum, are already blocked genetically. Additionally, the other afferent system, mossy fibres, as well as Purkinje cell axons themselves all feed into the very cerebellar nuclei that lidocaine is intended to block. Other drugs such as the GABA-A receptor agonist muscimol could also be used to eliminate cerebellar output in *Ptf1a*^*Cre*^*;Vglut2*^*fx/fx*^ mice. The inactivation would be different since lidocaine blocks additional cellular components. However, at the behavioural level, lidocaine and muscimol injections into the cerebellar nuclei produce similar consequences^67^,^68^,^69^. This equivalence likely exists because blocking the output of the nuclei determines the behavioural outcome, and therefore inhibiting passing axons has little consequence because they eventually feed into the nuclei that are blocked. Regardless, the lidocaine experiment results raised a therapeutic challenge. Which symptom is most problematic, and which one should (and can) be rescued to improve mobility?

Our model presented an opportunity to harness the endogenous potential of cerebellar circuits for functional brain repair of dystonia-like behaviours. We chose to use DBS because of its effectiveness in treating a wide range of neurological problems, including dystonia, Parkinson's disease, essential tremor, epilepsy, Tourette's syndrome and obsessive–compulsive disorder. Recent work even demonstrates improvement in cognitive function in a mouse model of Rett syndrome^35^. The improvement in mobility after DBS into the interposed cerebellar nuclei of the *Ptf1a*^*Cre*^*;Vglut2*^*fx/fx*^ mice (Supplementary Movie 8) opens the possibility of targeting the cerebellar network in different human motor and nonmotor brain diseases.

## Methods

### Animals

Dr Chris Wright (Vanderbilt University School of Medicine) kindly provided the *Ptf1a*^*Cre*^ mice^14^. We purchased the *Vglut2*^*floxed*^ (*Vglut2*^*fx*^, #012898) mice^70^ from The Jackson Laboratory (Bar Harbor, ME, USA) that contained *LoxP* sites surrounding exon 2 of the gene encoding vesicular glutamate transporter 2 (VGLUT2). *Rosa*^*lox-stop-lox-tdTomato*^ mice were purchased from The Jackson Laboratory (#007908) by Dr Benjamin R. Arenkiel (Baylor College of Medicine) and then kindly provided to us. *Neuropeptide Y* (*Npy-Gfp*) BAC transgenic mice were also purchased from The Jackson Laboratory (#008321). We bred the mice using timed pregnancies, and we designated noon on the day a vaginal plug was detected as embryonic day (E) 0.5 and the day of birth as P0. For the developmental stages of the study, mice of ages P0, 7, 9, 14 and 19–21 were used. For mature and adult studies, mice from P30 to P180 were used. Mice of both sexes were studied. All animal studies were carried out under an approved institutional animal care and use committee animal protocol according to the institutional guidelines at the Baylor College of Medicine.

### Tissue processing and anatomy

The mice were perfused with 1 M phosphate-buffered saline (PBS) and 4% paraformaldehyde (PFA) and the tissue cut on a cryostat or a microtome. Immunohistochemistry and *in situ* hybridization were carried out as described previously^28^. For *in situ* hybridization, mice were anaesthetized with isoflurane, brains rapidly removed from the skull and immersed in OCT (optimal cutting temperature), and immediately flash frozen by placing the tissue moulds into liquid nitrogen. Sagittal sections (25 μm) were cut through the cerebellum and the sectioned slices placed directly onto electrostatically coated glass slides (Probe On Plus Fisher Brand; Fisher Scientific). The tissue was probed with *Vglut2* (*SLC17A6*) or *Vglut1* (*SLC17A7*) digoxigenin-labelled mRNA probes using an automated *in situ* hybridization procedure termed Genepaint. All reagent incubations, washes and staining were performed by the *in situ* robot. The signal was detected by colourimetric detection using BCPI/NBT reagents. Upon completion of processing, the slides were removed from the machine and then cover-slipped with permanent mounting medium (Entellan mounting media (Electron Microscopy Sciences, Hatfield, PA, USA)). The mRNA *in situ* probes were designed against the sequences indicated below.

*Vglut2* (*SLC17A6*) sequence:

5′-CCAAATCTTACGGTGCTACCTCACAGGAGAATGGAGGCTGGCCTAACGGCTGGGAGAAAAAGGAAGAATTTGTGCAAGAAGGTGCGCAAGACGCGTACACCTATAAGGACCGAGATGATTATTCATAACGATGCTAGTTGCTGGATTCATTTGTAGTGTTTGTGAATCAATTAATTGTGATTGCACAAAAATAATTTTAAAAATGTGGTGTGAACATGTAAACATATCAACCAAGCAAGTCTTGCTGTTCAAAAACAAAAACAAAAAAATCTGAATTCAAAACAGACCATGAGATTCCCATCAAGTGCAATCTGTGGCAGTTGTCACGTTATGCCGTCTTCATTCAGGCCATTTGTCCTTTCGTTTGTGATTTAAAGGTTTCCTGTAGAAATAAGTAGGTATTCGTTGGACCCATCACCATTTTAGAGAGCACAACTACAACAGTTGGCACATGTCATCCTACAGAAGTTAGGAAGCCAAAGCTACTGGATCATGCAAACTGCACTTATTTATTACACTGGACTGCAAACTATCCCAGGGAAAGCCTGTCTAGAGACATAGTGGAACAGGAAAGATGGCT-3′.

*Vglut1* (*SLC17A7*) sequence:

5′-TGGGGATATTCAGGGAGGGGATACTGAGGTAAGCAAGCAAGTAGAACTGAGCTTCTAGGCAGCAGCGCCTGTAGCTAAAGTGGTAGGGCAGGCCCTAGCAACATAGAAGGGTGGAGCTCCACTGAGCTCTATTGGTAGATTTGGGCAGAGGGCGGGGCATCTTGTTGCTGATTGGCTAAGGAGCTAAGTCTATGGGTGCTGATTGGTAGAGGGTAGAGTCTGGGGGGCATCTAGTTTGATGTGTATTTTGACCC-3′.

For immunohistochemistry, free-floating sections were incubated in antibodies in a solution of 10% normal goat or donkey serum and 0.01% Tween-20. For DAB staining, sections were first bathed in H_2_O_2_ to quench endogenous peroxidases and then washed 3 times in PBS for 5 min each. For all stains, sections were blocked for 2 h with the blocking solution at room temperature and then incubated in primary antibodies overnight at room temperature. Sections were then washed with PBS 3 times for 5 min each and then incubated in secondary antibodies for 2 h. Sections were washed again 3 times in PBS for 5 min each and mounted onto slides. DAB slides were air-dried overnight and then dehydrated in ethanol before cover-slipping. Slides with fluorescent signal were immediately cover-slipped using FluoroGel as a medium. Antibodies used for immunohistochemistry (primary antibodies) were: cocaine- and amphetamine-related transcript (CART, rabbit (Rb), Santa Cruz, 1:500); calbindin (Rb and mouse (Ms), Swant, 1:10,000); carbonic anhydrase VIII (CAR8, Rb and Ms, Santa Cruz Biotechnology, Santa Cruz, CA, USA, 1:1,000 and 1:500, respectively); NeuN (Rb, Millipore, 1:500); VGLUT2 (Rb and Gp, Synaptic Systems; 1:1,000); VGLUT1 (Ms and Gp, Synaptic Systems, 1:1,000); VGAT (Rb and Gp, Synaptic Systems, 1:1,000); NFH (Ms, Covance, 1:1,500); TH (Rb, Millipore, 1:500); cFos (Rb, Calbiochem, 1:1,500); GFP (Ch, Abcam, 1:1,000); parvalbumin (Rb, Swant, 1:1,000); calretinin (Rb, Swant, 1:500); neurogranin (Rb, Chemicon, 1:500); Gamma-aminobutyric acid receptor α6 (GABARα6, Rb, Millipore, 1:500); and anti-lidocaine (Ms, Santa Cruz, 1:1,000). Secondary antibodies were: for the DAB reaction, we used horseradish peroxidase-conjugated goat anti-rabbit and goat anti-mouse secondary antibodies (diluted 1:200 in PBS; DAKO); staining for fluorescent immunohistochemistry was performed using donkey anti-mouse, anti-rabbit or anti-guinea pig secondary antibodies conjugated to Alexa 488, 555 or 647 fluorophores (Invitrogen), all diluted to 1:1,500. For DBS tissue, some sections were immunostained for cFos, and then they were lightly counterstained with haematoxylin. For electron microcopy, mice were perfused with 1% PFA and 1% glutaraldehyde. After perfusion, the cerebella were dissected and stored in fresh fixative overnight at 4 °C. Then, 0.5 to 1 mm sagittal sections of each cerebellum were post-fixed with 2% osmium tetroxide for 2–3 h, dehydrated through an ascending series of ethanol solutions and embedded in epon812. Ultrathin sections were cut, mounted on uncoated copper grids, stained with 2% uranyl acetate and 1% lead citrate for 12 min each. The tissue was examined using a JEOL 1010 microscope and the data imported into Adobe Photoshop CS5 for analysis.

### Analysis of stained images

Photomicrographs of tissue sections were captured using Zeiss AxioCam MRm (fluorescence) and AxioCam MRc5 (DAB-reacted tissue sections) cameras mounted on a Zeiss Axio Imager.M2 microscope or on a Zeiss Axio Zoom.V16. Images of tissue sections were acquired and analysed using either Zeiss AxioVision software (release 4.8) or Zeiss ZEN software (2012 edition). After imaging, the raw data were imported into Adobe Photoshop CS5 and corrected for brightness and contrast levels. Schematics were drawn in Adobe Illustrator CS5. For the full sagittal image shown in Supplementary Fig. 1, 10 × images were taken and then overlaid together in Adobe Photoshop CS5. Molecular layer thickness was determined by measuring in ImageJ the distance from the top of the Purkinje cell soma to the top of the Purkinje cell dendritic tree. Measurements were taken from four mice of each genotype, from each of the four cerebellar transverse zones. Age-matched, gender-matched mice of the same genotype were averaged and were compared using Student's *t*-test. ImageJ was used for counting for the recombination efficiency experiments. Recombination efficiency in the inferior olive was determined by driving expression of tdTomato in the olive using the *Ptf1a*^*Cre*^ driver line. The olive was also stained for NeuN. The images were then separated by colour, thresholded and using the manual counting feature in ImageJ, the number of tdTomato-positive cells and the number of NeuN-positive cells were counted. A percentage was calculated using tdTomato-positive/NeuN-positive × 100. For the analysis of protein puncta in the molecular layer and cerebellar nuclei, tissue sections were stained with calbindin (to label Purkinje cells) and VGLUT2, NFH (to label neurons of the cerebellar nuclei and determine the borders of the nuclei) and VGLUT2 or NFH and VGAT. Images were separated by colour, auto-contrasted, auto-thresholded, run through a built-in watershed and counted using the analysed particles feature. The number of puncta/area were averaged and compared between genotypes using Student's *t*-test. Fluorescence intensity values were acquired in ImageJ by finding the mean pixel intensity in a region of interest. For WGA-Alexa 555 (diluted to 1% in PBS pH 7.4; Thermo Fisher Scientific, Waltham, MA, USA) marking of the interposed cerebellar nuclei after recording, ∼10–20 nl of the tracer was injected and then the mice perfused (as described above) after recording. The tissue was cryoprotected, cut at 40 μm, mounted, and the fluorescence signal imaged directly. The Alexa 555 signal was overlaid on brightfield images of the same tissue.

### Behavioural analyses

Rotarod performance was quantified by recording the latency to fall or to rotate 3 consecutive times on an accelerating rod (Ugo Basile, Varese, Italy). Mice were tested on the rotarod each day for 3 days with 3 trials separated by 15 min. Tremor amplitude and frequency were analysed on a single trial with a Tremor Monitor^28^ (San Diego Instruments, San Diego, CA, USA). Using the provided software from SDI, the raw waveforms were passed through a fast Fourier transform to create a power spectrum graph. The power spectrum was then condensed to 1 Hz bins and averaged across all mice and graphed with error bars indicating s.e.m. For statistical analysis, the peak, centred at 9–13 Hz, was compared by Student's *t*-test. The open field activity assay was carried out using an Open Field Locomotion system (Omnitech Electronics, Inc., Columbus, OH, USA). Mice were acclimated to the Open Field room for 30 min with white noise provided. Mice were then placed in the Open Field apparatus and tracked for 30 min. Between-group differences were statistically evaluated by Student's *t*-test. Videos were taken using a Sony 9.2 megapixel HDR-PJ540 camera. Videos were then processed to demonstrate the motor behaviours with PlayMemories and iMovie software. Mice were weighed at several time points and averaged for each age, for each genotype. The data at each time point were gender-matched and then compared with an unpaired Student's *t*-test.

### Lidocaine infusion

Mini-osmotic pumps (Alzet, Cupertino, CA, USA) were primed and filled with 4% lidocaine (Sigma-Aldrich, L7757) diluted in 0.9% saline with 0.01% methylene blue dye or just 0.9% saline with 0.01% methylene blue dye as a sham control. The pumps were connected to custom-built cannulas (Plastics One, Roanoke, VA, USA) that were targeted to release the lidocaine^71^, which blocks cerebellar firing *in vivo*^72^, to the interposed nuclei for 7 days. Tremor was measured before, during and after treatment (see below). Mice were perfused for anatomical examination shortly after the pump was depleted, but after the final tremor analysis was conducted to verify the surgical targeting of the cannulas. Intrasubject data were tested using a paired *t*-test and inter-subject data were tested using an unpaired *t*-test.

### Deep brain stimulation

Twisted bipolar electrodes were fabricated out of 50 μm tungsten wire. The spacing between the two electrodes was calculated to bilaterally target the interposed cerebellar nuclei or the centrolateral nuclei of the thalamus. We used a Master 8 pulse generator and an Iso-Flex stimulus isolator (AMPI, Jerusalem, Israel) to deliver a biphasic 130-Hz stimulus with 60 μs duration at 30 μA for 1 h per day for 5 days. Mice were filmed throughout the process. The videos were cut into regular interval 1 min segments, randomized and then analysed in a blinded manner using a dystonia rating scale^3^,^73^ before, during and after DBS. The 5 min of video was analysed for each period, with the ‘during DBS' period starting immediately after the stimulator was turned on and with the ‘after/post-DBS' period starting immediately after the stimulator was shut off. Dystonia rating scale from 0 to 5 as in Pizoli *et al*.^73^ was used wherein 0=no motor abnormalities; 1=slightly slowed or abnormal motor behaviour, no dystonia; 2=mild impairment, sometimes limited ambulation, dystonic postures when disturbed; 3=moderate impairment, frequent spontaneous dystonic postures; 4=severe impairment, sustained dystonic postures and limited ambulation; and 5=prolonged immobility in dystonic postures. Mutant mouse ratings were examined using a paired *t*-test or one-way repeated-measures ANOVA. Implanted but nonstimulated mice were used as sham controls. Mice were perfused after the final day of testing to examine the cerebellum for anatomical verification of surgical targeting of the electrodes. For the cFos experiments the mice were perfused with 4% PFA, as described above, 1 h after DBS.

### *In vivo* electrophysiology

For the anaesthetized *in vivo* recordings of Purkinje cells, inhibitory interneurons and cerebellar nuclei neurons, the mice were given ketamine/dexmedetomidine (75 and 0.5 mg kg^−1^, respectively) and maintained with ∼0.25% isoflurane^28^. For head-fixed, awake recordings, mice were implanted with custom-made headplates and a craniotomy made above the cerebellum^56^,^74^. After 72 h of recovery, the mice were trained for 30 min per day in a head-fixed apparatus for 3 days before recording^56^,^74^. Cells were recorded and categorized based on standard stereotaxic coordinates measured from Bregma^75^. Control Purkinje cells were identified by the presence of complex spike activity. Analysis of complex spike percentage was taken from the awake recorded and juxtacellular labelled cells, as well from metal electrode recorded cells that were held briefly in anaesthetized mice. We examined the presence or absence of a complex spike for a total of 47 control Purkinje cells and 119 mutant Purkinje cells. For both awake and anaesthetized metal electrode recording procedures, single-unit recordings were attained with 5–8 MΩ tungsten electrodes (Thomas Recording, Germany) and then the signals digitized into Spike2 (CED, England) where single units were verified with principal component analysis. Analysis of continuous traces of >300 s was performed with Spike2, MS Excel and MATLAB (Mathworks, Natick, MA, USA). We calculated the total number of simple and complex spikes over a predefined period of recording to obtain simple and complex spike firing frequency (Hz=spikes s^−1^). Firing pattern variability, or regularity, is defined as a measure of the consistency of time intervals between spikes (interspike time interval or ISI=seconds). To quantify the average variability in firing pattern, CV of the ISI was calculated as the ratio of the s.d. of ISIs to the mean ISI of a given cell. We measured local regularity with CV2. CV2 measures firing pattern variability within a short period of two interspike intervals (CV2=2|ISI_*n*+1_–ISI_*n*_|/(ISI_*n*+1_+ISI_*n*_)^76^. Spike rates, CV and CV2 were computed and reported as mean±s.e.m. Statistical analyses were performed with unpaired, two-tailed Student's *t*-tests or one-way ANOVA. Developmental Purkinje cell recordings were compared using nonparametric statistical analyses with the Kruskall–Wallis test, followed by Dunn's multiple comparison test for *post hoc* analyses between the two groups. Statistical significance is indicated in the graphs as *P<0.05, **P<0.01 or ***P<0.001.

### Juxtacellular recording

We labelled Purkinje cells in anaesthetized 3–5-month-old mice using 8–10 MΩ glass electrodes that were filled with 1% neurobiotin (Vector Laboratories Inc., Burlingame, CA, USA) diluted in 0.9% sterile saline. After identifying and then entraining a single unit, we performed a loose-patch fill^77^ by delivering 1–5 nA of positive current for 45–60 min. The mice were then perfused and the neurobiotin labelling of single filled cells detected using the VECTASTAIN Elite ABC method (Vector Laboratories Inc.) and visualized with DAB.

### ECoG and EMG recordings

For ECoG, 00-90 × 1/16 flat point stainless steel machine screws (#B002SG89QQ) were implanted into the skull over the cerebellum (−6.9 mm from Bregma at the midline) and cerebral cortex (−1 mm anterior–posterior from Bregma, 1 mm medial–lateral). For EMG, 76.2 μm silver wire was implanted into the gastrocnemius muscle and the coated portion of the wire directed below the skin into custom-built ports (also receives EEG connections; Pinnacle Technology, Lawrence, KS, USA) that were secured to the skull. We used a custom-built Pinnacle Technology ECoG plus EMG pre-amplifier to amplify the muscle and brain signals before further amplification with a Model 410 Brownlee amplifier. ECoG and EMG signals were recorded for 15 min in each mouse. The signals were digitized into Spike2. Kainic acid (Sigma-Aldrich, St Louis, MO, USA) was delivered once per hour at a dosage of 5 mg kg^−1^ to induce seizure activity. The ECoG signal during visually apparent seizure activity was compared with the ECoG during either normal locomotion or dystonia, all normalized to the baseline (quiescent) ECoG signal.

### Data availability

Data from the experiments presented in the current study are available from the corresponding author on request.

## Additional information

**How to cite this article:** White, J. J. & Sillitoe, R. V. Genetic silencing of olivocerebellar synapses causes dystonia-like behaviour in mice. *Nat. Commun.* **8,** 14912 doi: 10.1038/ncomms14912 (2017).

**Publisher's note:** Springer Nature remains neutral with regard to jurisdictional claims in published maps and institutional affiliations.

## Supplementary Material

Supplementary Information Supplementary Table and Supplementary Figures

Supplementary Movie 1 Movie comparing the posture and limb motions of Vglut2^fx/fx^ and Ptf1a^Cre^;Vglut2^fx/fx^ pups. Dystonia-like behaviors are evident in mutant mice as early as postnatal day 7. The same pups were examined at P10 and P14 to follow the persistence of dystonia-like motor behaviors.

Supplementary Movie 2 Movie of a control compared to an adult Ptf1a^Cre^;Vglut2^fx/fx^ mouse. The mutant exhibits extreme dystonic postures that include hyperextension of the limbs and toes, twisting of the torso (also see movie of pups Supplementary Movie 1). and rigid posturing of the limbs and back.

Supplementary Movie 3 Movie of an adult Ptf1a^Cre^;Vglut2^fx/fx^mouse. The general ambulatory movements of the animal are jerky, although even with this impaired mobility they are able to explore the cage and perform fundamental behaviors such as eating and mating. But as activity proceeds, the mice display severe bouts of limb and torso hyperextension as well as twisting postures. The toes of the extended limbs are splayed apart.

Supplementary Movie 4 ECoG/EMG recording from a Vglut2^fx/fx^ mouse during normal locomotion. A control mouse exploring its enclosure after implantation of ECoG electrodes (cerebellum and cerebral cortex) and EMG electrodes (gastrocnemius).

Supplementary Movie 5 ECoG/EMG recording from a Vglut2^fx/fx^ mouse during kainate-induced seizure Kainic acid injections induce seizure-like activity that is associated with abnormal movements in adult mice.

Supplementary Movie 6 ECoG/EMG recording from a Ptf1a^Cre^;Vglut2^fx/fx^ mouse taken during the periods of overt dystonic postures that are observed in the mutants. Ptf1aCre;Vglut2^fx/fx^ mice exhibit dystonia-like postures that are distinct from seizure-like movements.

Supplementary Movie 7 Lidocaine infusion into the cerebellum. A movie featuring clips of the same mutant mouse before, during, and after lidocaine infusion targeted to the interposed cerebellar nuclei.

Supplementary Movie 8 Deep brain stimulation (DBS) of the cerebellum. A movie featuring clips of Vglut2^fx/fx^ and Ptf1a^Cre^;Vglut2^fx/fx^ mice before, during, and after targeting deep brain stimulation to the interposed cerebellar nuclei.

Supplementary Movie 9 Deep brain stimulation (DBS) of the centrolateral nucleus of the thalamus. A movie featuring clips of a Ptf1a^Cre^;Vglut2^fx/fx^ mouse before and during deep brain stimulation of thecentrolateral nucleus of the thalamus, which connects the cerebellum to the basal ganglia.
