# Supplementary material for: Genetic silencing of olivocerebellar synapses causes dystonia-like behaviour in mice
Source: Nat Commun. 2017 Apr 4;8:14912. doi: 10.1038/ncomms14912 (PMC5382291; doi:10.1038/ncomms14912)
Supplement: Supplementary Information — Supplementary Table and Supplementary Figures [file ncomms14912-s1.pdf]

**Table 1**	Genotype	Quantification	*n*	*p*-value	Related Figure
VGLUT2 ML puncta	$Vglut2^{fx/fx}$	$163 \pm 7.74/10^4\ \mu m^2$	4 mice	9.294×10^{-16}	Fig. 1f
	$Ptf1a^{Cre};Vglut2^{fx/fx}$	$14 \pm 2.1/10^4\ \mu m^2$	4 mice		
VGLUT2 mossy fiber terminals	$Vglut2^{fx/fx}$	$112 \pm 6.6/10^4\ \mu m^2$	4 mice	0.705	Fig. 1g
	$Ptf1a^{Cre};Vglut2^{fx/fx}$	$114 \pm 18.10/10^4\ \mu m^2$	4 mice		
VGLUT1 intensity	$Vglut2^{fx/fx}$	54 ± 3.57	4 mice	0.594	Supplementary Fig. 3a
	$Ptf1a^{Cre};Vglut2^{fx/fx}$	57 ± 4.30	4 mice		
Rotarod time to fall (third day)	$Vglut2^{fx/fx}$	236 ± 9.83 s	62 mice	1.746×10^{-14}	Fig. 3c
	$Ptf1a^{Cre};Vglut2^{fx/fx}$	80 ± 13.41 s	38 mice		
OFA total distance	$Vglut2^{fx/fx}$	3200 ± 249 cm	28 mice	9.62×10^{-4}	Fig. 3e
	$Ptf1a^{Cre};Vglut2^{fx/fx}$	2100 ± 184 cm	29 mice		
OFA distance/ activity count	$Vglut2^{fx/fx}$	0.57 ± 0.02	28 mice	7.20×10^{-6}	Fig. 3e
	$Ptf1a^{Cre};Vglut2^{fx/fx}$	0.39 ± 0.02	29 mice		
Tremor frequency	$Vglut2^{fx/fx}$	12.3 ± 0.708 Hz	139 mice	0.705	Fig. 3f
	$Ptf1a^{Cre};Vglut2^{fx/fx}$	11 ± 1.14 Hz	129 mice		
Tremor power	$Vglut2^{fx/fx}$	$2.1 \times 10^{-4} \pm 2.92 \times 10^{-5}$	139 mice	1.05×10^{-6}	Fig. 3f
	$Ptf1a^{Cre};Vglut2^{fx/fx}$	$5.6 \times 10^{-4} \pm 3.98 \times 10^{-5}$	129 mice		
Juvenile anesthetized PC frequency	$Vglut2^{fx/fx}$	26 ± 4.95 Hz	7 cells from 2 mice	4.1×10^{-2}	Fig. 4c
	$Ptf1a^{Cre};Vglut2^{fx/fx}$	15 ± 4.99 Hz	12 cells from 3 mice		
Juvenile anesthetized PC CV	$Vglut2^{fx/fx}$	2.86 ± 3	7 cells from 2 mice	3.7×10^{-3}	Fig. 4c
	$Ptf1a^{Cre};Vglut2^{fx/fx}$	0.443 ± 0.099	12 cells from 3 mice		
Juvenile anesthetized PC CV2	$Vglut2^{fx/fx}$	0.46 ± 0.05	7 cells from 2 mice	0.593	Fig. 4c
	$Ptf1a^{Cre};Vglut2^{fx/fx}$	0.37 ± 0.08	12 cells from 3 mice		
Adult anesthetized PC frequency	$Vglut2^{fx/fx}$	66 ± 4.5 Hz	27 cells from 8 mice	0.548	See text
	$Ptf1a^{Cre};Vglut2^{fx/fx}$	62 ± 5.4 Hz	45 cells from 14 mice		
Adult anesthetized PC CV	$Vglut2^{fx/fx}$	0.568 ± 0.049	27 cells from 8 mice	0.852	See text
	$Ptf1a^{Cre};Vglut2^{fx/fx}$	0.555 ± 0.051	45 cells from 14 mice		
Adult anesthetized PC CV2	$Vglut2^{fx/fx}$	0.44 ± 0.021	27 cells from 8 mice	0.751	See text
	$Ptf1a^{Cre};Vglut2^{fx/fx}$	0.45 ± 0.022	45 cells from 14 mice		
Adult anesthetized MLIs frequency	$Vglut2^{fx/fx}$	19.6 ± 2.99 Hz	11 cells from 5 mice	0.545	Supplementary Fig. 5c
	$Ptf1a^{Cre};Vglut2^{fx/fx}$	17.3 ± 2.33 Hz	17 cells from 6 mice		
Adult anesthetized MLIs CV	$Vglut2^{fx/fx}$	1.13 ± 0.279	11 cells from 5 mice	0.846	Supplementary Fig. 5c
	$Ptf1a^{Cre};Vglut2^{fx/fx}$	1.07 ± 0.143	17 cells from 6 mice		
Adult anesthetized MLIs CV2	$Vglut2^{fx/fx}$	0.565 ± 0.053	11 cells from 5 mice	0.376	Supplementary Fig. 5c
	$Ptf1a^{Cre};Vglut2^{fx/fx}$	0.639 ± 0.062	17 cells from 6 mice		

Adult awake PC frequency	*Vglut2*$^{fx/fx}$	75 ± 6.21 Hz	18 cells from 7 mice	0.695	Fig. 4h
	Ptf1a^{Cre};*Vglut2*$^{fx/fx}$	72 ± 5.35 Hz	17 cells from 11 mice		
Adult awake PC CV	*Vglut2*$^{fx/fx}$	0.56 ± 0.05	18 cells from 7 mice	0.622	Fig. 4h
	Ptf1a^{Cre};*Vglut2*$^{fx/fx}$	0.60 ± 0.05	17 cells from 11 mice		
Adult awake PC CV2	*Vglut2*$^{fx/fx}$	0.45 ± 0.03	18 cells from 7 mice	0.204	Fig. 4h
	Ptf1a^{Cre};*Vglut2*$^{fx/fx}$	0.50 ± 0.02	17 cells from 11 mice		
P7 ML thickness	*Vglut2*$^{fx/fx}$	51 ± 1.2 μm	4 mice	2.3×10^{-4}	Fig. 5
	Ptf1a^{Cre};*Vglut2*$^{fx/fx}$	39 ± 1.5 μm	4 mice		
Adult ML thickness	*Vglut2*$^{fx/fx}$	147.2 ± 2.5 μm	5 mice	0.069	Fig. 5
	Ptf1a^{Cre};*Vglut2*$^{fx/fx}$	145.6 ± 4.3 μm	5 mice		
Juvenile anesthetized CN frequency	*Vglut2*$^{fx/fx}$	3.11 ± 0.71 Hz	8 cells from 3 mice	1.35×10^{-2}	Fig. 6f
	Ptf1a^{Cre};*Vglut2*$^{fx/fx}$	14.9 ± 3.9 Hz	11 cells from 3 mice		
Juvenile anesthetized CN CV	*Vglut2*$^{fx/fx}$	2.25 ± 0.42	8 cells from 3 mice	0.98	Fig. 6f
	Ptf1a^{Cre};*Vglut2*$^{fx/fx}$	2.26 ± 0.65	11 cells from 3 mice		
Juvenile anesthetized CN CV2	*Vglut2*$^{fx/fx}$	0.86 ± 0.06	8 cells from 3 mice	0.053	Fig. 6f
	Ptf1a^{Cre};*Vglut2*$^{fx/fx}$	0.66 ± 0.07	11 cells from 3 mice		
Adult awake CN frequency	*Vglut2*$^{fx/fx}$	54 ± 4.4 Hz	33 cells from 14 mice	3.594×10^{-5}	Fig. 6i
	Ptf1a^{Cre};*Vglut2*$^{fx/fx}$	28 ± 3.6 Hz	27 cells from 10 mice		
Adult awake CN CV	*Vglut2*$^{fx/fx}$	0.52 ± 0.04	33 cells from 14 mice	5.6×10^{-5}	Fig. 6i
	Ptf1a^{Cre};*Vglut2*$^{fx/fx}$	1.08 ± 0.12	27 cells from 10 mice		
Adult awake CN CV2	*Vglut2*$^{fx/fx}$	0.42 ± 0.03	33 cells from 14 mice	6.56×10^{-4}	Fig. 6i
	Ptf1a^{Cre};*Vglut2*$^{fx/fx}$	0.64 ± 0.05	27 cells from 10 mice		

Supplementary Table 1. Data for anatomical, behavioral and *in vivo* electrophysiological quantifications.

A table containing data for the anatomical, behavioral and *in vivo* electrophysiological characterizations of *Ptf1a*^{Cre}*;Vglut2*$^{fx/fx}$ mutant mice compared to *Vglut2*$^{fx/fx}$ control mice. All p-values are from the unpaired Student's t-test. Abbreviations: ML = molecular layer, OFA = open field analysis, PC = Purkinje cell, CV = coefficient of variation of the ISIs, MLIs = molecular layer interneurons, CN = cerebellar nuclei.

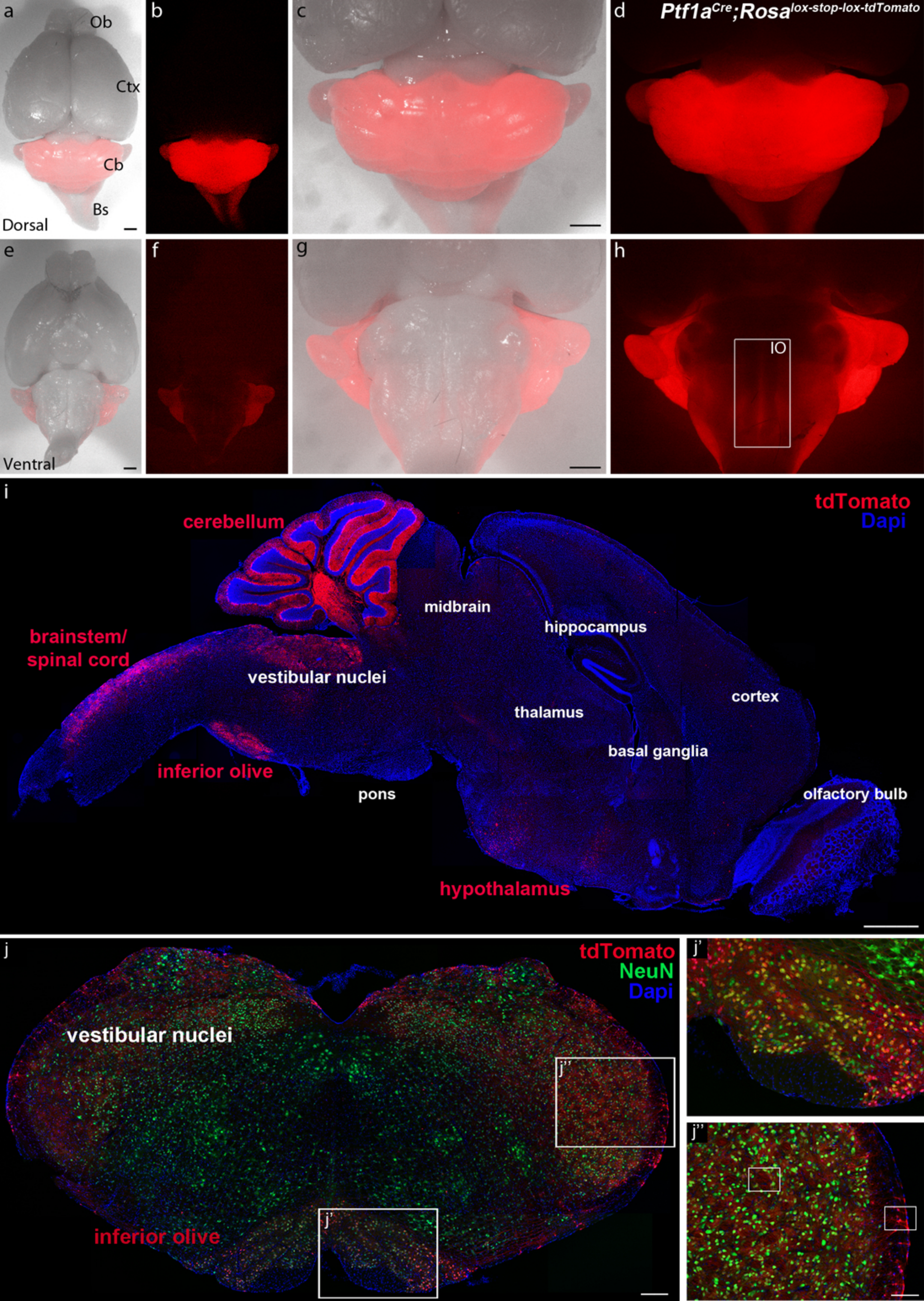

a
Ob
Ctx
Cb
Bs
Dorsal
b
c
d
Ptf1a^Cre;Rosa^lox-stop-lox-tdTomato
e
Ventral
f
g
h
IO
i
tdTomato
Dapi
cerebellum
midbrain
hippocampus
brainstem/
spinal cord
vestibular nuclei
thalamus
cortex
basal ganglia
inferior olive
pons
olfactory bulb
hypothalamus
j
tdTomato
NeuN
Dapi
vestibular nuclei
inferior olive
j'
j''

Supplementary Figure 1. ***Ptf1a^{Cre}*** **drives heavy tdTomato expression in the hindbrain circuitry.**

(a-h) Whole mount views of a *Ptf1a^{Cre};Rosa$^{lox\text{-}stop\text{-}lox\text{-}tdTomato}$* mouse brain with fluorescent images overlaid on bright field images in (**a, c, e, g**). Abbreviations: Ob = olfactory bulb, Ctx = cortex, Cb = cerebellum, Bs = brainstem, IO = inferior olive. Scale bars = 1 mm.

(i) Shows a sagittal brain section that was assembled into a montage from higher power images from a *Ptf1a^{Cre};Rosa$^{lox\text{-}stop\text{-}lox\text{-}tdTomato}$* mouse. Areas with strong tdTomato labeling in somata are labeled in red, all other brain structures are labeled in white. Scale bar = 500 μm.

(j) Brainstem section demonstrating overlap between *Ptf1a-Cre* driven tdTomato expression and NeuN expression. Overlap in the somata is observed in the inferior olive, labeled in red in (**j**), and higher power is shown in (**j'**), while other areas with tdTomato expression, such as adjacent to the vestibular nucleus (labeled with white letters in (**j**)), do not overlap with NeuN expression but are rather in non-neural cells (small box on right) and passing fibers (small box on left) as seen in (**j''**). Scale bar in **j** = 200 μm and scale bar in **j"** = 100 μm.

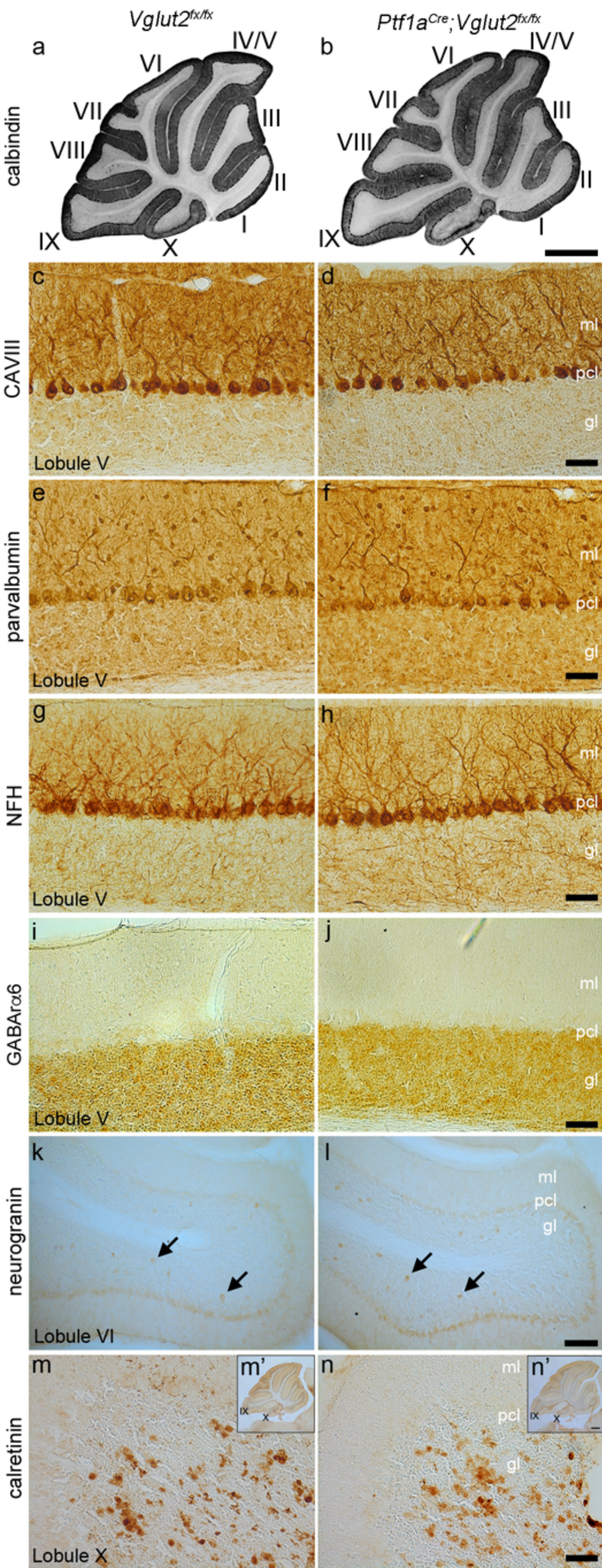

Vglut2fx/fx
Ptf1aCre;Vglut2fx/fx
a
b
calbindin
IV/V
VI
VII
VIII
IX
X
III
II
I
c
d
CAVIII
ml
pcl
gl
Lobule V
e
f
parvalbumin
Lobule V
g
h
NFH
Lobule V
i
j
GABARα6
Lobule V
k
l
neurogranin
Lobule VI
m
m'
n
n'
calretinin
Lobule X

Supplementary Figure 2. The molecular expression patterns of cellular markers and the size of the cerebellum are intact in the *Ptf1a*^{Cre}*;Vglut2*$^{fx/fx}$ mutant mice.

(a and b) Foliation and size of the cerebellum are normal in *Ptf1a*^{Cre}*;Vglut2*$^{fx/fx}$ mice as compared to *Vglut2*$^{fx/fx}$ littermates. Scale bar = 2 mm.

(c and d) Purkinje cells are labeled by carbonic anhydrase VIII (CAVIII) and their position is normal in the adult cerebellum. Scale bar = 50 µm.

(e and f) Expression of the GABAergic marker parvalbumin labels Purkinje cells and molecular layer interneurons with no difference between *Vglut2*$^{fx/fx}$ and *Ptf1a*^{Cre}*;Vglut2*$^{fx/fx}$ mice. Scale bar = 50 µm.

(g and h) Neurofilament heavy (NFH) labels a subset of Purkinje cells and basket cell axons. The termination of basket cells axons on Purkinje cells is intact. Scale bar = 50 µm.

(i and j) Expression of GABAalpha6, which labels differentiated granule cells in the granular layer, is normal. Scale bar = 50 µm.

(k and l) Golgi cells heavily express neurogranin (arrows) and reside in the granular layer as normal. Scale bar = 100 µm.

(m and n) Unipolar brush cells in lobule X express calretinin as normal. Scale bar = 50 µm. In the *Ptf1a*^{Cre}*;Vglut2*$^{fx/fx}$ mutants, unipolar brush cells are restricted mainly to lobules IX and X as expected and are therefore not ectopically located beyond their typical circuit, **m'** and **n'**. Scale bar = 500 µm. Abbreviations: ml = molecular layer, pcl = Purkinje cell layer, gl = granular layer.

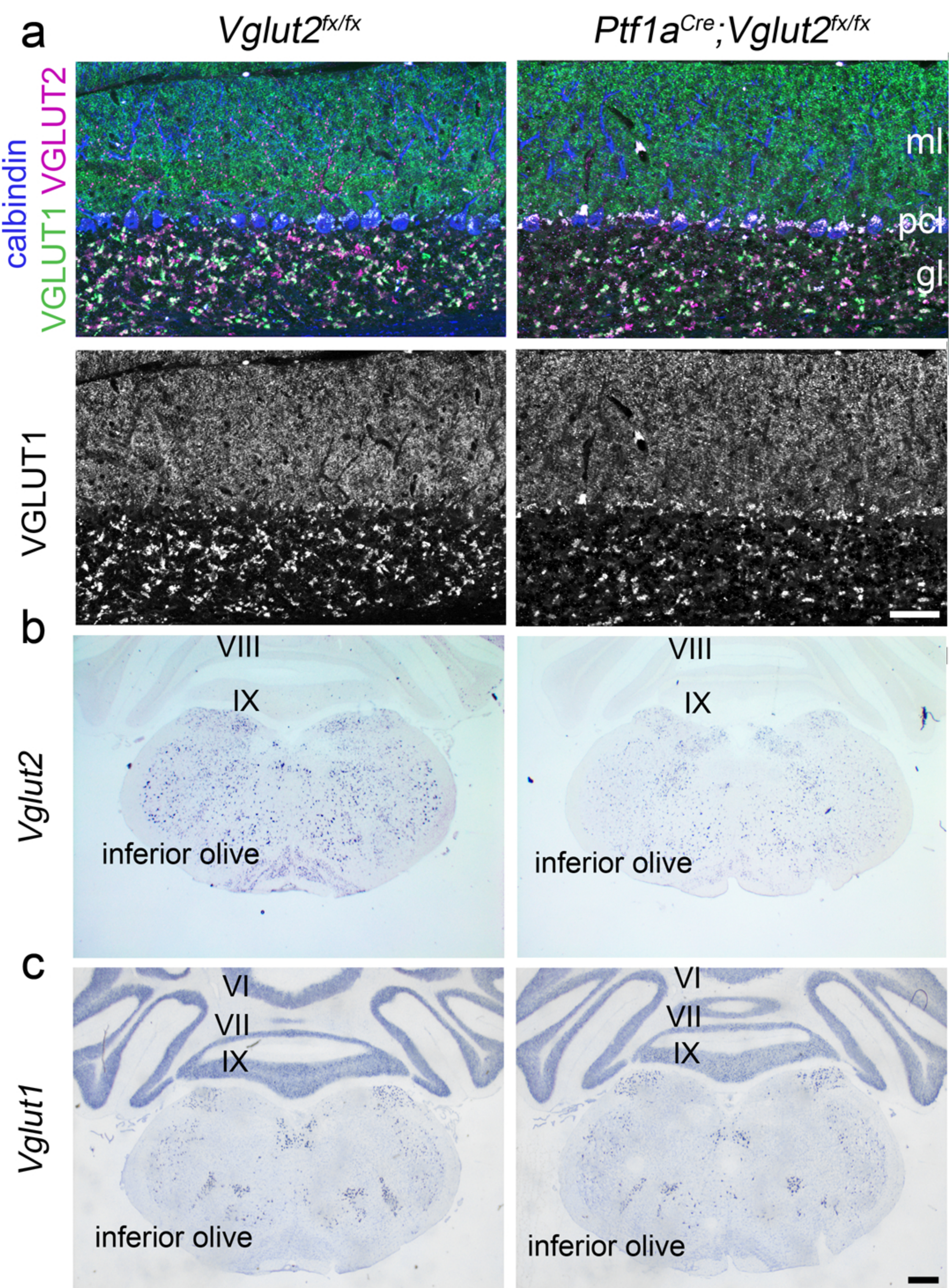

a
Vglut2fx/fx
Ptf1aCre;Vglut2fx/fx
calbindin VGLUT1 VGLUT2
ml
pcl
gl
VGLUT1
b
VIII
IX
Vglut2
inferior olive
VIII
IX
inferior olive
c
VI
VII
IX
Vglut1
inferior olive
VI
VII
IX
inferior olive

Supplementary Figure 3. *Vglut1* does not compensate for the loss of *Vglut2* in the inferior olive.

(a) Expression of VGLUT1, which is normally localized to parallel fiber terminals in the molecular layer, remained unchanged after the removal of VGLUT2 ($p = 0.594$)**.** $n > 3$ mice per genotype. Scale bar = 50 µm.

(b) Genetic deletion of *Vglut2* as assessed by the depletion of mRNA in the inferior olive of the adult mutant mouse.

(c) *Vglut1* was not upregulated in the inferior olive after conditional deletion of *Vglut2*. Scale bar in **c** = 500 µm (also applies to **b**). Abbreviations: ml = molecular layer, pcl = Purkinje cell layer, gl = granular layer.

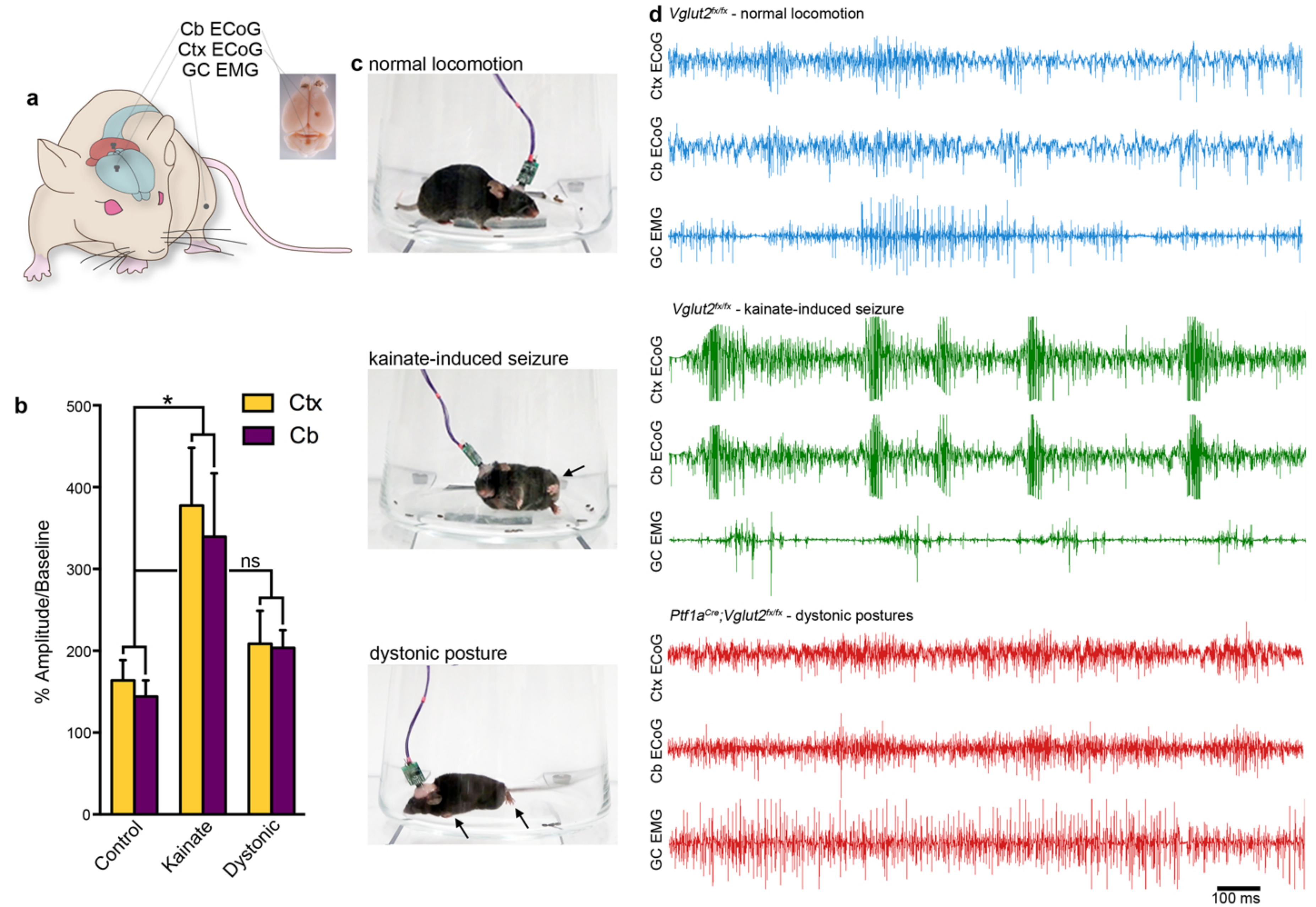

a
Cb ECoG
Ctx ECoG
GC EMG
b
% Amplitude/Baseline
500
400
300
200
100
0
*
ns
Ctx
Cb
Control
Kainate
Dystonic
c normal locomotion
kainate-induced seizure
dystonic posture
d Vglut2fx/fx - normal locomotion
Ctx ECoG
Cb ECoG
GC EMG
Vglut2fx/fx - kainate-induced seizure
Ctx ECoG
Cb ECoG
GC EMG
Ptf1aCre;Vglut2fx/fx - dystonic postures
Ctx ECoG
Cb ECoG
GC EMG
100 ms

Supplementary Figure 4. Loss of neurotransmission from the inferior olive to the cerebellum does not cause seizure-like activity.

(a) Schematic example of recording sites for ECoG and EMG electrodes as well as a brain image showing localization of ECoG electrodes. Schematic adapted with permission, from drawings published in ref. 74.

(b) Quantification of amplitude of the ECoG signal normalized to baseline. While a significant difference was found between kainate-induced seizure and normal locomotion (during exploratory behavior) (ctx: p = 0.0184, cb: p = 0.0187), no significant difference was found between control, normal locomotion and the dystonic $Ptf1a^{Cre};Vglut2^{fx/fx}$ mice (ctx: p = 0.4047, p = cb: 0.0892). *n* = 5 mice in all conditions. Student's unpaired t-test.

(c) Still images of mice in each condition. Arrows point to abnormal postures.

(d) Sample ECoG and EMG traces from each condition. Scale bar = 100 ms.

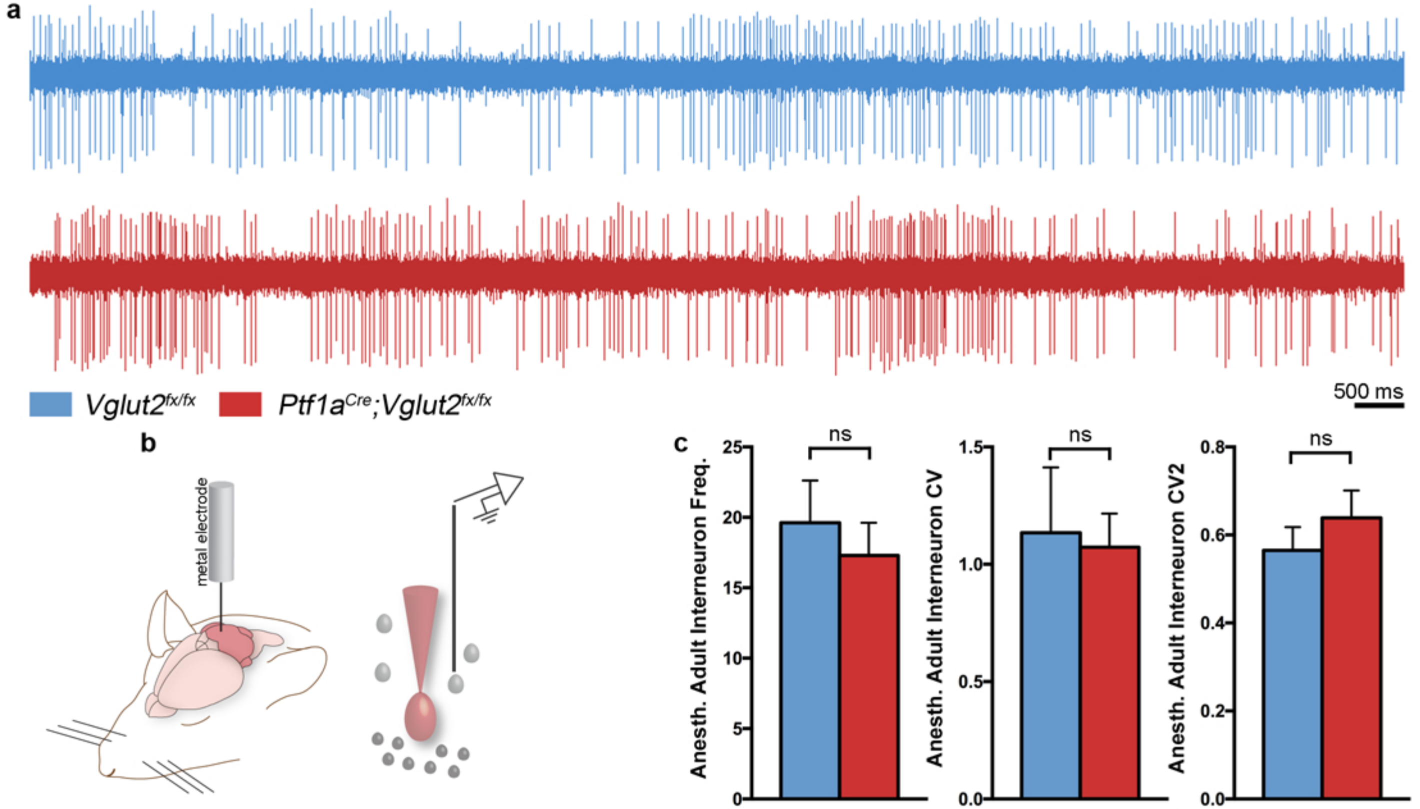
a
Vglut2fx/fx
Ptf1aCre;Vglut2fx/fx
500 ms
b
metal electrode
c
ns
Anesth. Adult Interneuron Freq.
0
5
10
15
20
25
Anesth. Adult Interneuron CV
0.0
0.5
1.0
1.5
Anesth. Adult Interneuron CV2
0.0
0.2
0.4
0.6
0.8

Supplementary Figure 5. Loss of olivocerebellar signaling does not affect the firing properties of cerebellar cortical interneurons.

(a) Single-unit extracellular recordings of putative stellate and basket cell inhibitory interneurons from superficial aspects of the cerebellum (no deeper than 1.5 mm) accessed from a craniotomy made over lobule VI in anesthetized mice. The recordings reveal very similar firing characteristics between interneurons of *Vglut2*$^{fx/fx}$ (top) and *Ptf1a*^{Cre};*Vglut2*$^{fx/fx}$ (bottom) mice. Scale bar = 500 ms.

(b) Schematic of the anesthetized cerebellar recording and electrode proximity to inhibitory interneurons in the molecular layer of the cerebellar cortex. Schematic adapted with permission, from drawings published in ref. 28 and 74.

(c) Firing properties were not significantly different between interneurons in *Vglut2*$^{fx/fx}$ and *Ptf1a*^{Cre};*Vglut2*$^{fx/fx}$ mice (n = 11 cells from 5 animals and 17 cells from 6 animals, respectively) (Frequency: $p = 0.545$; CV: $p = 0.846$; CV2: $p = 0.376$). Error bars are defined as s.e.m. Student's unpaired t-test.

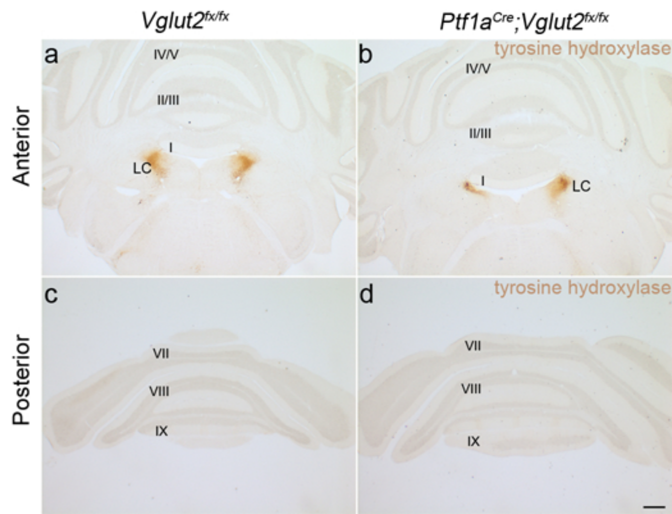

Vglut2fx/fx
Ptf1aCre;Vglut2fx/fx
Anterior
Posterior
a
b
c
d
tyrosine hydroxylase
tyrosine hydroxylase
IV/V
II/III
I
LC
IV/V
II/III
I
LC
VII
VIII
IX
VII
VIII
IX

Supplementary Figure 6. Tyrosine Hydroxylase is not upregulated in *Ptf1a*^{Cre}*;Vglut2*$^{fx/fx}$ mutant mice.

(a - d) Tyrosine hydroxylase (TH) is often expressed ectopically in the Purkinje cells of adult mutant mice with altered cerebellar function[28]. TH expression is not upregulated in the adult *Ptf1a*^{Cre}*;Vglut2*$^{fx/fx}$ mutant mouse cerebellum in either anterior (shown: lobules I, II/III, IV/V) or posterior sections (shown: lobules VII, VIII, IX). TH expression in the locus coeruleus (LC) was not changed in *Ptf1a*^{Cre}*;Vglut2*$^{fx/fx}$ mice. $n > 4$ mice per genotype. Scale bar = 500 µm.

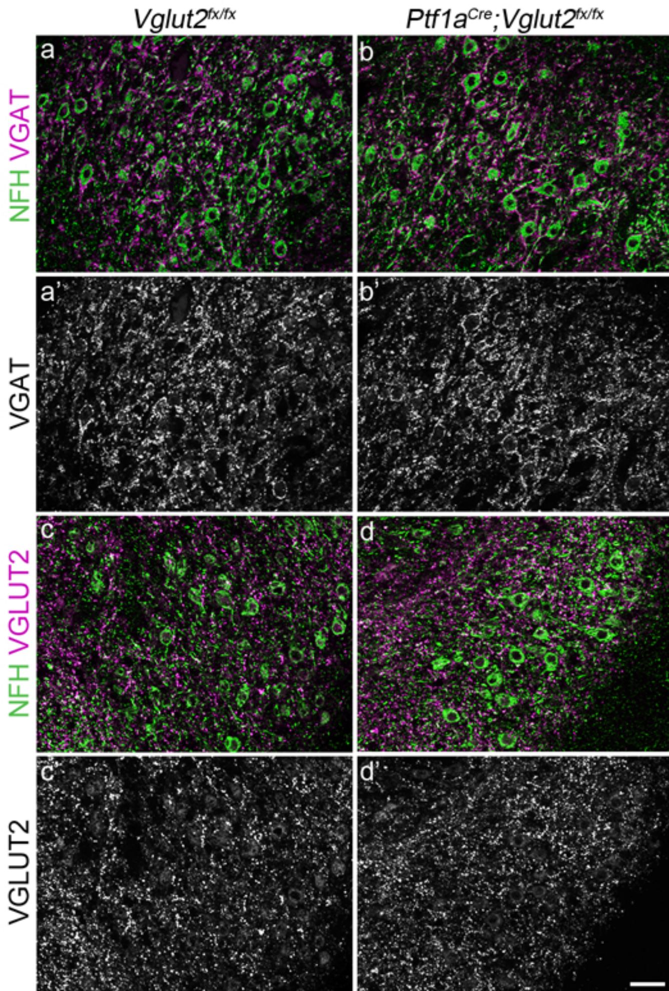

Vglut2fx/fx
Ptf1aCre;Vglut2fx/fx
a
b
NFH VGAT
a'
b'
VGAT
c
d
NFH VGLUT2
c'
d'
VGLUT2

Supplementary Figure 7. There was no significant difference in VGAT and a minor difference in VGLUT2 expression in the cerebellar nuclei after loss of VGLUT2 in axon terminals that project from the inferior olive.

(a, a', b and b') Analysis of VGAT expression in the cerebellar nuclei of *Vglut2*$^{fx/fx}$, compared to *Ptf1a*^{Cre}*;Vglut2*$^{fx/fx}$ mice, revealed similar numbers of puncta between the genotypes (*Vglut2*$^{fx/fx}$ = 3480 ± 90.5, *n* = 4; *Ptf1a*^{Cre}*;Vglut2*$^{fx/fx}$ = 3500 ± 140.7, *n* = 7; p = 0.765; Student's unpaired t-test). **(c, c', d, and d')** Analysis of VGLUT2 expression in the cerebellar nuclei of *Vglut2*$^{fx/fx}$ mice, compared to *Ptf1a*^{Cre}*;Vglut2*$^{fx/fx}$ mice, revealed a small, but significant decrease in the number of puncta between the genotypes, likely representing the loss of VGLUT2 in the olivocerebellar collaterals that project to the cerebellar nuclei (*Vglut2*$^{fx/fx}$ = 3300 ± 120.5, *n* = 5; *Ptf1a*^{Cre}*;Vglut2*$^{fx/fx}$ = 2800 ± 173.1; p = 0.0229, *n* = 6, Student's unpaird t-test). Scale bar = 50 µm.

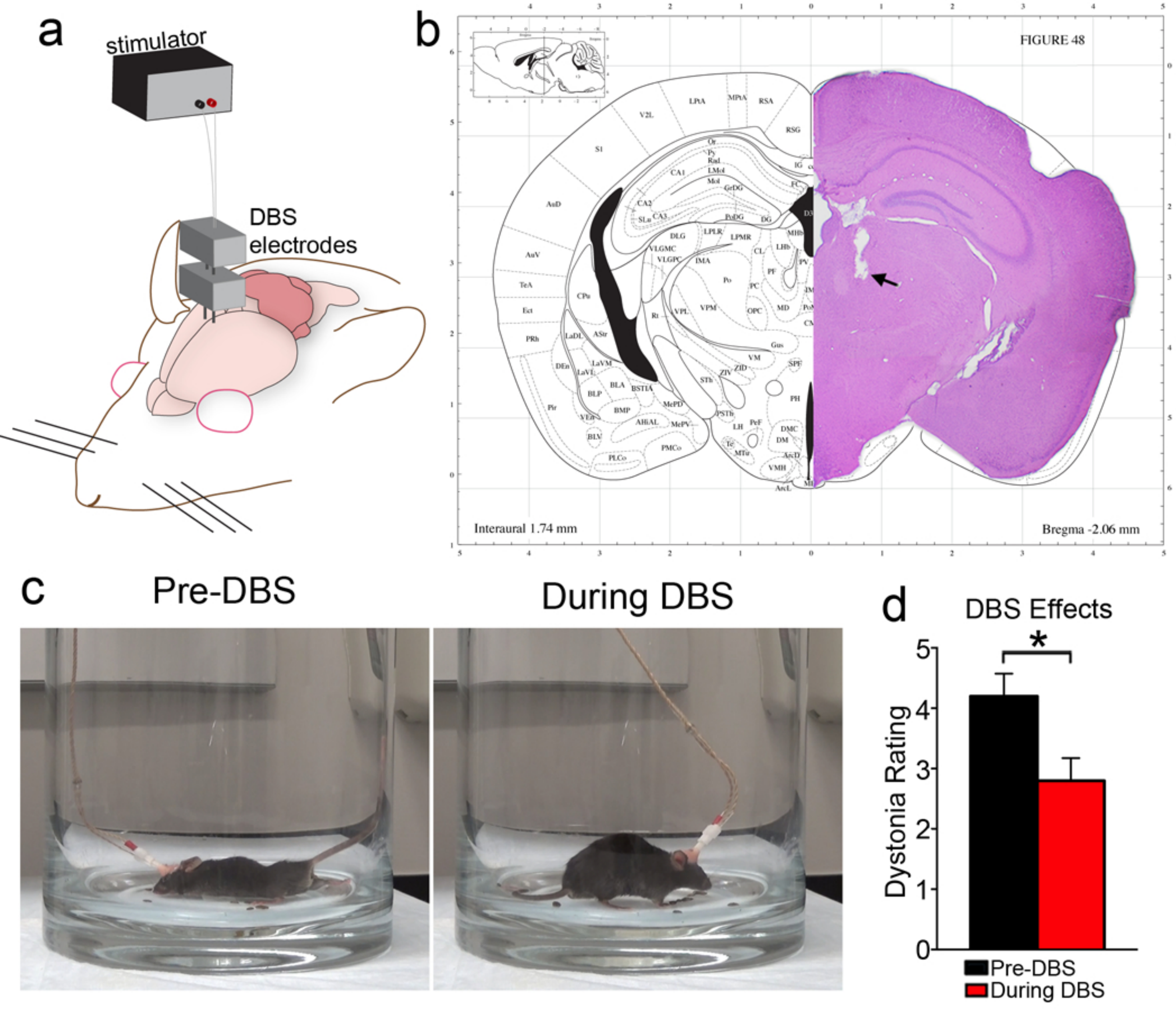
a
stimulator
DBS
electrodes
b
FIGURE 48
Interaural 1.74 mm
Bregma -2.06 mm
c
Pre-DBS
During DBS
d
DBS Effects
*
Dystonia Rating
0
1
2
3
4
5
Pre-DBS
During DBS

Supplementary Figure 8. Deep brain stimulation (DBS) of the centrolateral nuclei of the thalamus recovers motor function in *Ptf1a*^{Cre}*;Vglut2*$^{fx/fx}$ mice.

(a) A schematic example of deep brain stimulation targeting the thalamus. Schematic adapted with permission, from drawings published in ref. 28.

(b) Histological confirmation of targeting the DBS electrodes to the centrolateral nuclei (arrow). Schematic reproduced in part, with permission, from Paxinos, G. & Franklin, K. B. J. *The Mouse Brain in Stereotaxic Coordinates* (2004).

(c) Stills of a *Ptf1a*^{Cre}*;Vglut2*$^{fx/fx}$ mouse before and during stimulation.

(d) Dystonia rating before and during DBS stimulation of the centrolateral thalamic nuclei in naïve *Ptf1a*^{Cre}*;Vglut2*$^{fx/fx}$ mutant mice ($p = 0.0249$, $n = 5$ mice, Student's paired t-test). Error bars are defined as s.e.m.
